# Low-Temperature Conversion of Discarded Cigarette Filters into Magnetic Porous Carbon for Diethyl Phthalate Removal

**DOI:** 10.3390/molecules31213979

**Published:** 2026-11-04

**Authors:** Wang-De Lin, Ru-Hau Ye, Hua-Wei Chen, Chia-Hsuan Yang, Gui-Bing Hong

**Affiliations:** 1 Department of Cosmetic Application and Management, St. Mary’s Junior College of Medicine, Nursing and Management, Yilan City 266, Taiwan; 2 Department of Chemical Engineering and Biotechnology, National Taipei University of Technology, Taipei City 106, Taiwan; 3 Department of Chemical and Materials Engineering, National Ilan University, Yilan City 260, Taiwan; 4 Office of Student Success Analytics, James Madison University, Harrisonburg, VA 22807, USA

**Keywords:** adsorption, cellulose acetate, cigarette filters, diethyl phthalate, magnetic modification

## Abstract

Discarded cigarette filters represent a widespread cellulose-acetate waste stream with the potential for conversion into functional carbon materials. In this study, discarded cigarette filters were converted into magnetic porous carbon for diethyl phthalate (DEP) adsorption through hydrothermal carbonization, H_3_PO_4_ activation at 300 °C, and subsequent Fe(II)/Fe(III) coprecipitation. Activation increased the Brunauer–Emmett–Teller (BET) specific surface area of the hydrochar from 5.96 to 312.47 m^2^ g^−1^, while magnetic modification further increased it to 542.41 m^2^ g^−1^. The incorporation of Fe-containing species was confirmed by X-ray photoelectron spectroscopy, and magnetic functionality was demonstrated by superconducting quantum interference device measurements and separation using an external magnet. DEP removal by the resulting material (MAC-11) was influenced by the initial DEP concentration, solution pH, adsorbent dosage, and temperature. Response surface methodology identified a candidate operating condition of 30.4 °C, 14.6 mg adsorbent, and 236 min, under which an independent confirmation experiment achieved 87.7% DEP removal, compared with a model prediction of 85.0%. The pseudo-second-order model provided a better empirical description of the adsorption kinetics than the pseudo-first-order model, while both the Langmuir and Freundlich models described the concentration-dependent adsorption data. Although magnetic modification reduced DEP adsorption relative to the nonmagnetic activated carbon (AC-300), MAC-11 retained appreciable adsorption performance while acquiring magnetic separability. These findings demonstrate the feasibility of converting discarded cigarette filters into magnetically responsive porous carbon through comparatively low-temperature activation, highlighting the trade-off between adsorption performance and magnetic recoverability.

## 1. Introduction

Cigarette butts (CBs) are a widespread form of anthropogenic litter and pose persistent environmental risks because of their extensive disposal and potential release of hazardous constituents [1,2,3]. Cigarette filters consist predominantly of cellulose acetate and may retain nicotine, metals, and other tobacco-derived contaminants that can subsequently be released into the environment [2,3,4]. Nevertheless, their polymeric composition also offers opportunities for resource recovery. Recent studies have investigated the conversion of discarded cigarette filters into value-added materials, including cellulose acetate-based membranes [4] and porous carbons [5,6,7]. Among these approaches, the production of carbonaceous adsorbents represents a promising strategy for valorizing cigarette-filter waste while developing functional materials for environmental remediation.

Hydrothermal carbonization (HTC) provides a relatively mild thermochemical route for converting carbon-containing wastes into hydrochar, which can serve as a precursor for activated carbon production [8,9,10]. Hydrochar and hydrochar-derived materials have been investigated for diverse applications, including solid fuels, soil amendments, and adsorbents for environmental remediation [10,11]. Subsequent chemical or physical activation can improve their textural properties, particularly specific surface area and pore accessibility, thereby enhancing their potential for water treatment [12,13,14,15]. Previous studies have demonstrated the potential of discarded cigarette filters as precursors for porous carbons, including activated carbons for U(VI) removal [5], high-surface-area carbons for hydrogen storage [6], and microspherical activated carbons for ammonia adsorption [7]. Among chemical activation methods, H_3_PO_4_ activation is particularly relevant to cellulose-based precursors because it promotes dehydration, structural transformation, and pore development during thermal treatment [12,13,14,15,16]. Notably, Fan et al. [16] demonstrated that substantial porosity could be developed through H_3_PO_4_ activation of cellulose at temperatures as low as 300 °C. However, discarded cigarette filters consist primarily of cellulose acetate rather than pristine cellulose, and their chemical composition and structure may be further altered during HTC. Therefore, whether a sufficiently developed and accessible pore structure can be obtained from cigarette-filter-derived hydrochar under similarly mild activation conditions remains to be established.

Diethyl phthalate (DEP) is a low-molecular-weight phthalate ester widely used as a plasticizer and as a solvent or fixative in fragrances and personal care products. With a molecular weight of 222.24 g mol^−1^ and an aqueous solubility of approximately 1080 mg L^−1^ at 25 °C, DEP can partition into the aqueous phase following environmental release. Because DEP is generally not chemically bound to the polymer matrices in which it is incorporated, it may migrate from DEP-containing products during use and disposal. Municipal wastewater and industrial effluents therefore represent potential pathways for its introduction into aquatic environments. Puri et al. [17] reported mean DEP concentrations of 2.25 and 0.77 μg L^−1^ in the influent and effluent, respectively, of an urban wastewater treatment plant, demonstrating that DEP may persist in treated wastewater. Although DEP is susceptible to biodegradation under favorable conditions, its detection in treated effluent indicates that wastewater discharge remains a potential pathway for environmental exposure. Furthermore, Kim et al. [18] reported alterations in locomotor activity, sleep patterns, and circadian rhythm-related gene expression in zebrafish larvae following experimental DEP exposure. These findings support continued investigation of DEP removal from contaminated water, although the adverse effects observed under laboratory exposure conditions should not be directly extrapolated to typical environmental concentrations.

Adsorption has been widely investigated for removing DEP and related phthalate esters from aqueous solutions because of its operational simplicity and the availability of diverse adsorbent materials. Activated carbon [19], waste-derived biochars [20], and porous polymeric adsorbents [21] have demonstrated the potential for DEP removal. However, adsorption performance depends not only on specific surface area but also on pore accessibility, pore-size distribution, and surface chemistry. Understanding how these characteristics influence DEP uptake is therefore important for developing effective waste-derived carbon adsorbents. An additional practical challenge is the recovery of fine adsorbent particles from treated water. Incorporating Fe-containing magnetic phases into carbonaceous materials can facilitate solid–liquid separation under an external magnetic field [22,23,24]. Magnetic adsorbents have also been investigated for the removal of phthalate esters from aqueous solutions [25], supporting the feasibility of combining contaminant removal with magnetic recovery. Nevertheless, magnetic modification may alter the pore structure, surface chemistry, and composition of the parent carbon, potentially affecting its adsorption performance. Consequently, enhanced magnetic separability does not necessarily correspond to improved adsorption capacity. Balancing adsorption performance and magnetic recoverability is therefore an important consideration in developing reusable carbon-based adsorbents for water treatment.

In this study, discarded cellulose acetate cigarette filters were converted into magnetic porous carbon through hydrothermal carbonization, H_3_PO_4_ activation, and subsequent magnetic modification. The primary objective was to investigate whether hydrochar derived from discarded cigarette filters could develop sufficient porosity for effective diethyl phthalate (DEP) adsorption at a relatively low activation temperature of 300 °C while retaining magnetic responsiveness for separation under an external magnetic field. The preparation conditions were evaluated, and the physicochemical properties of the resulting carbon materials were characterized to examine the effects of activation and magnetic modification. The influence of operating variables on DEP removal was investigated, and response surface methodology was applied to evaluate their individual and interactive effects and identify favorable operating conditions. Adsorption kinetics and equilibrium behavior were analyzed using nonlinear model fitting, and the adsorption performance of the prepared materials was compared with that of commercial activated carbons. This study provides insights into the relationships among low-temperature activation, material properties, DEP adsorption behavior, and magnetic separability, contributing to the development of waste-derived carbon adsorbents for water treatment.

## 2. Results and Discussion

### 2.1. Preparation-Condition Screening

The effectiveness of ethanol pretreatment was assessed by comparing discarded cigarette filters before and after cleaning using inductively coupled plasma optical emission spectrometry (ICP-OES) and UV–Vis spectroscopy (Figure S1). The ICP-OES results indicated decreases in the measured concentrations of several elements following ethanol washing, suggesting a reduction in selected inorganic constituents associated with the discarded filters. Similarly, the UV–Vis spectra showed a pronounced decrease in absorbance in the approximately 330–450 nm region after cleaning, consistent with a reduction in UV-absorbing constituents. Together, these results suggest that ethanol pretreatment reduced certain measurable contaminants. However, these analyses do not establish the complete removal of nicotine, polycyclic aromatic hydrocarbons, plastic additives, or other potentially hazardous organic compounds. The pretreatment results should therefore be interpreted as evidence of partial contaminant reduction rather than comprehensive decontamination.

**Figure 1:**
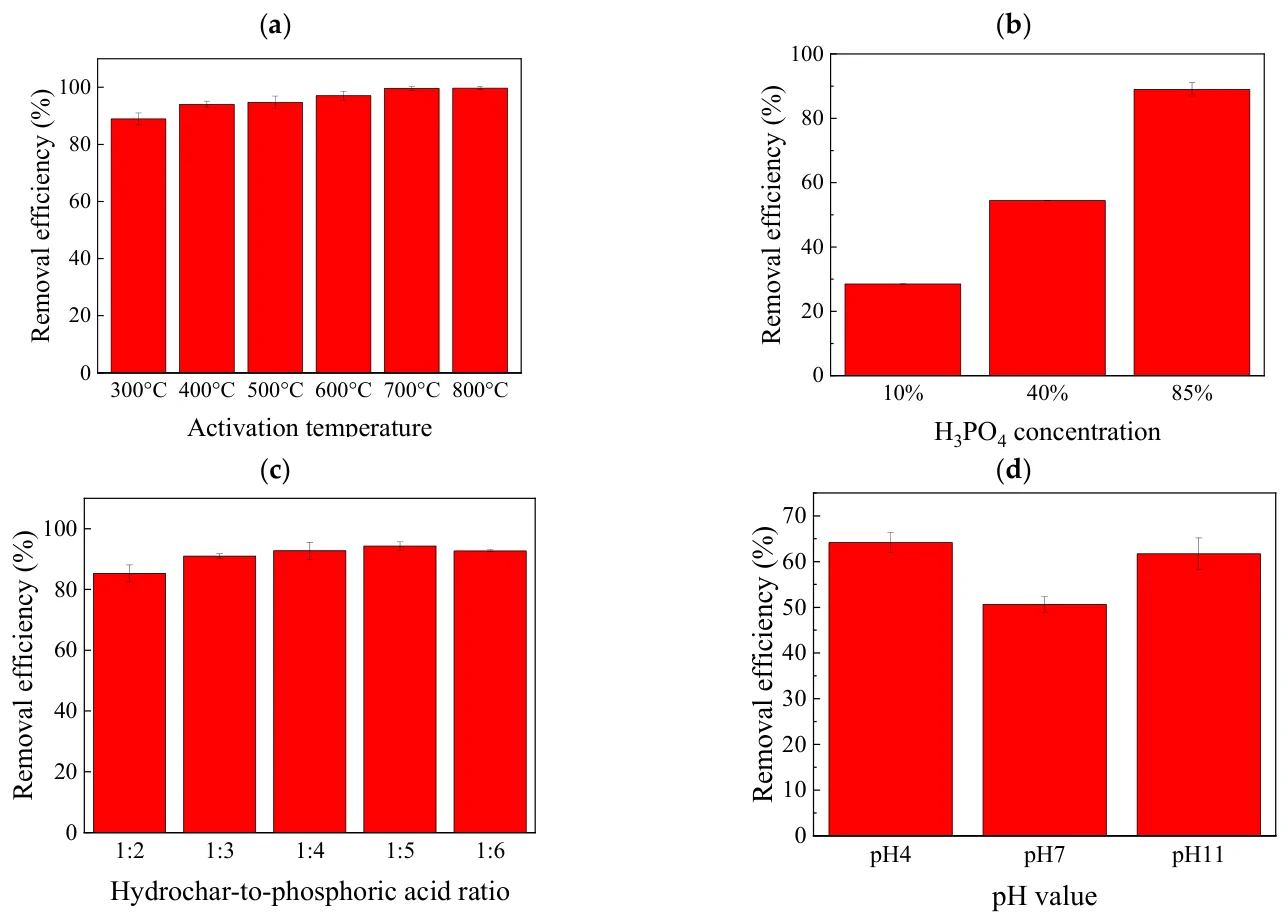
Effects of preparation conditions on DEP removal by cigarette-filter-derived porous carbons: (**a**) activation temperature, (**b**) H_3_PO_4_ concentration, (**c**) HTC-CB-to-H_3_PO_4_ solution mass ratio, and (**d**) pH during magnetic modification. Adsorption screening conditions: initial DEP concentration, 100 mg L^−1^; solution volume, 20 mL; adsorbent dosage, 10 mg; temperature, 30 °C; shaking speed, 100 rpm; and contact time, 24 h. Error bars represent the standard deviations of three independent adsorption experiments (n = 3).

The effects of activation temperature, H_3_PO_4_ concentration, HTC-CB-to-H_3_PO_4_ solution mass ratio, and magnetic-modification pH on DEP removal are presented in Figure 1. Activation temperature substantially influenced the adsorption performance of the cigarette-filter-derived activated carbons (Figure 1a). Increasing the activation temperature from 300 to 800 °C increased DEP removal from 89.0% to 99.7%. Nevertheless, AC-300 already achieved approximately 90% removal under the screening conditions. Therefore, 300 °C was selected for subsequent experiments, not because it provided the highest removal efficiency but to investigate the feasibility of producing an effective adsorbent from cigarette-filter-derived hydrochar at a comparatively low activation temperature.

Previous studies have demonstrated that activation temperature and H_3_PO_4_ loading strongly influence pore development and surface chemistry during the chemical activation of carbonaceous precursors [12,13,14,15,16]. H_3_PO_4_ can promote dehydration, crosslinking, structural rearrangement, and pore formation during thermal treatment [12,13,14,15]. Notably, Fan et al. [16] demonstrated that H_3_PO_4_ activation of cellulose at 300 °C could produce activated carbon with substantial porosity. However, their study employed cellulose as the precursor, whereas the present study used hydrochar derived from discarded cellulose-acetate cigarette filters. Accordingly, the significance of the present results lies not in establishing 300 °C H_3_PO_4_ activation as a new process, but in demonstrating that substantial DEP removal can be achieved using a waste-derived hydrochar activated under this relatively mild thermal condition.

H_3_PO_4_ concentration strongly affected DEP removal at 300 °C (Figure 1b). Increasing the acid concentration from 10 to 85 wt% improved DEP removal from 28.5% to 89.0%. This improvement may be associated with changes in pore development and surface chemistry during activation, as suggested by previous studies [12,13,14,15,16]. However, because the materials prepared at different H_3_PO_4_ concentrations were not individually characterized for their pore structures, the contribution of specific textural changes cannot be established. Among the concentrations examined, 85 wt% H_3_PO_4_ yielded the highest DEP removal and was selected for subsequent experiments. The effect of the HTC-CB-to-H_3_PO_4_ solution mass ratio was less monotonic (Figure 1c). DEP removal increased with increasing H_3_PO_4_ solution loading, reached its highest value at a mass ratio of 1:5, and then decreased slightly at 1:6. Nevertheless, a ratio of 1:3 already achieved a removal efficiency of 90.1%. Although changes in activating-agent loading can influence pore development and surface functionality [12,13,14,15,16], the present screening data do not identify the specific factors responsible for the differences in DEP removal. Therefore, the 1:3 ratio was selected as a practical compromise between activating-agent consumption and adsorption performance, avoiding the higher H_3_PO_4_ solution loadings required at ratios of 1:4–1:6.

Magnetic modification was subsequently performed on AC-300 through Fe(II)/Fe(III) coprecipitation. In contrast to the preceding screening experiments involving nonmagnetic activated carbons, Figure 1d presents the DEP removal performance of the magnetically modified materials. Their generally lower removal efficiencies may reflect changes in adsorption-site accessibility or surface chemistry following the incorporation of Fe-containing phases. However, the available data do not allow these contributions to be quantified. DEP removal also varied nonmonotonically with the initial pH used during magnetic modification, with MAC-7 exhibiting lower removal than MAC-4 and MAC-11 (Figure 1d). Differences in the formation and deposition of Fe-containing species may have contributed to this behavior. Nevertheless, because the materials prepared at different modification pH values were not characterized comparatively, the specific cause of the lower DEP removal by MAC-7 remains uncertain. MAC-11 was selected for subsequent experiments based on its DEP adsorption performance and observed magnetic responsiveness, as further discussed in Section 3.2.

Overall, the screening results supported the selection of an activation temperature of 300 °C, an H_3_PO_4_ solution concentration of 85 wt%, an HTC-CB-to-H_3_PO_4_ solution mass ratio of 1:3, and an initial magnetic-modification pH of 11. These conditions were selected to establish a comparatively low-temperature preparation route while maintaining substantial DEP removal and providing magnetic functionality rather than to maximize each preparation variable independently.

### 2.2. Physicochemical Characteristics of the Prepared Carbons

**Figure 2:**
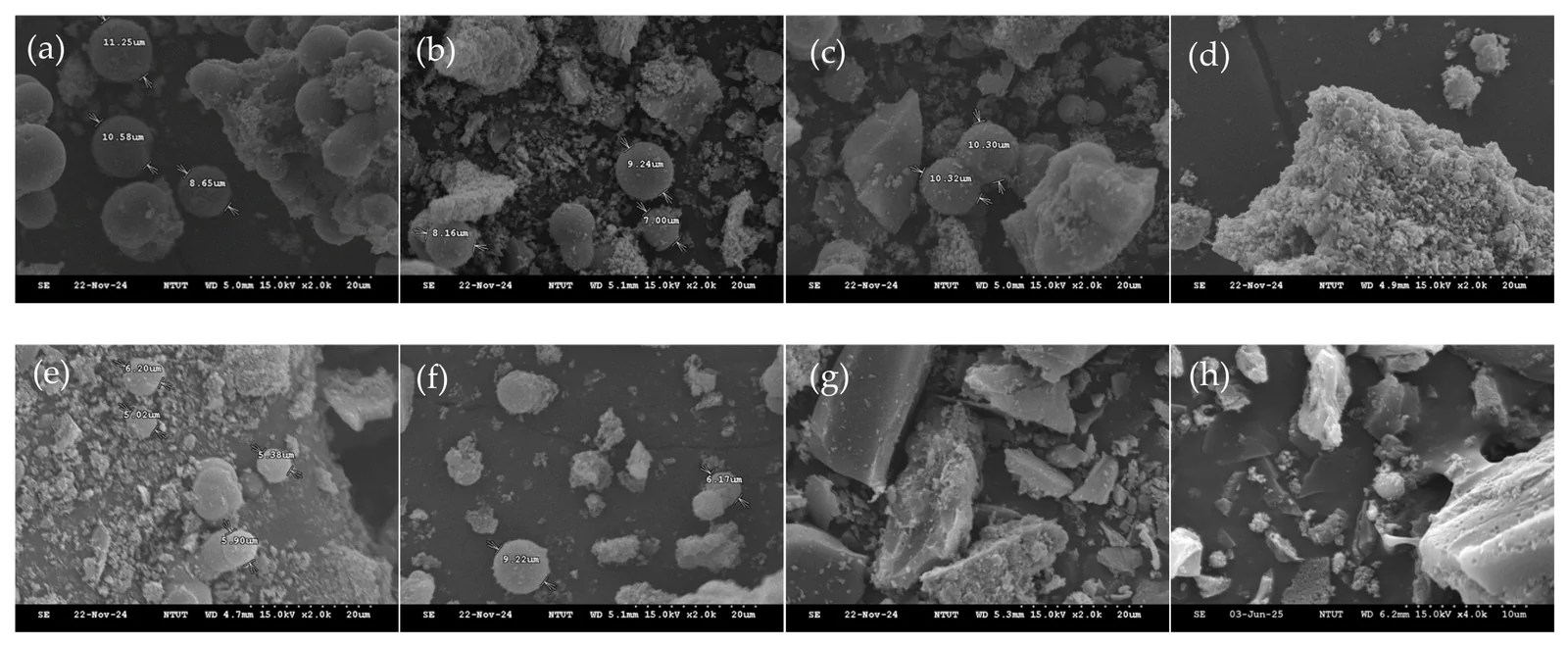
SEM images of cigarette-filter-derived porous carbons prepared at different activation temperatures and commercial activated carbons: (**a**) AC-300, (**b**) AC-400, (**c**) AC-500, (**d**) AC-600, (**e**) AC-700, (**f**) AC-800, (**g**) CAC-1, and (**h**) CAC-2.

The surface morphologies of the cigarette-filter-derived carbons and commercial activated carbons are presented in Figure 2. AC-300 exhibited a heterogeneous surface characterized by numerous spherical or sphere-like features. As the activation temperature increased, these features became less distinct, and the carbon matrix appeared increasingly irregular and fragmented. In contrast, the commercial activated carbons displayed different surface morphologies, likely reflecting differences in their precursor materials and preparation processes. These observations suggest that thermal activation induced substantial morphological changes in the cigarette-filter-derived carbons. However, SEM observations alone cannot establish the presence or development of micro- and mesopores relevant to DEP adsorption; these textural characteristics are more appropriately assessed using N_2_ adsorption–desorption analysis, as discussed below.

The bulk elemental compositions of HTC-CB, AC-300, MAC-11, CAC-1, and CAC-2 are summarized in Table 1. The measured carbon content decreased from 64.61% in HTC-CB to 49.34% in AC-300 following H_3_PO_4_ activation and further to 30.69% in MAC-11 after magnetic modification. By comparison, CAC-1 and CAC-2 exhibited substantially higher carbon contents of 80.94% and 76.09%, respectively. The lower carbon mass fraction of MAC-11 is consistent with the incorporation of inorganic Fe-containing species during coprecipitation. Nevertheless, because the elemental analysis did not quantify oxygen, phosphorus, or iron, the measured compositions do not provide a complete elemental mass balance. Accordingly, the observed differences in carbon content should be interpreted as variations in bulk elemental composition rather than direct evidence of differences in carbonization degree.

**Figure 3:**
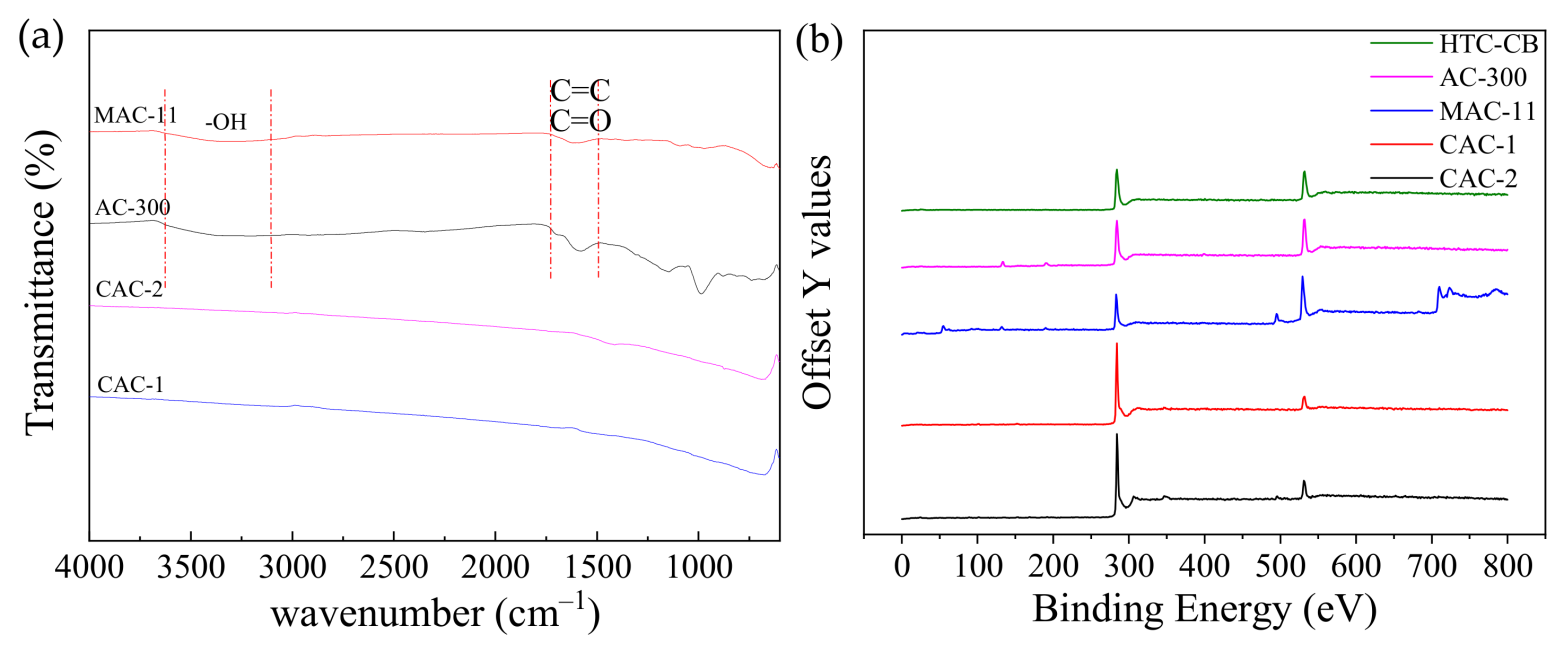
Surface chemical characteristics of cigarette-filter-derived and commercial activated carbons: (**a**) FTIR spectra and (**b**) XPS survey spectra.

The FTIR spectra revealed differences in surface functional groups among the prepared and commercial activated carbons (Figure 3a). AC-300 and MAC-11 exhibited broad absorption bands at approximately 3400–3500 cm^−1^, primarily associated with O–H stretching vibrations. Absorption features in the 1600–1700 cm^−1^ region may be attributed to overlapping contributions from C=O stretching vibrations and aromatic C=C skeletal vibrations. The presence of these spectral features in AC-300 suggests that oxygen-containing functional groups remained detectable after activation at 300 °C, consistent with previous reports describing the temperature-dependent evolution of surface functionalities during H_3_PO_4_ activation of cellulosic precursors [12,13,14,15,16]. In comparison, the commercial activated carbons exhibited less pronounced absorption features in these regions, indicating differences in their detectable surface functional groups. However, these qualitative spectral differences do not provide a direct measure of the relative abundance of individual functional groups.

The XRD patterns of the prepared materials are presented in Figure S2. The broad diffraction features observed for the carbonaceous samples are consistent with predominantly disordered carbon structures. For MAC-11, the diffraction pattern did not contain sufficiently distinct reflections to allow reliable identification of a specific crystalline iron-oxide phase. Therefore, the Fe-containing component was not assigned to Fe_3_O_4_ or another particular iron-oxide phase solely on the basis of the XRD results.

The XPS survey spectra provide evidence of Fe incorporation into MAC-11 following magnetic modification (Figure 3b). The analyzed carbonaceous samples exhibited prominent C 1s and O 1s signals at approximately 284 and 533 eV, respectively, whereas MAC-11 additionally displayed Fe 2p signals at approximately 711 and 722 eV. These observations confirm the presence of Fe-containing species within the XPS sampling depth of MAC-11. However, because only survey spectra were available, the Fe oxidation states could not be reliably distinguished. Moreover, XPS survey spectra do not establish crystalline phase identity. Accordingly, the combined XRD and XPS results support the incorporation of Fe-containing species but do not provide sufficient evidence to identify the magnetic component as a specific iron-oxide phase.

**Figure 4:**
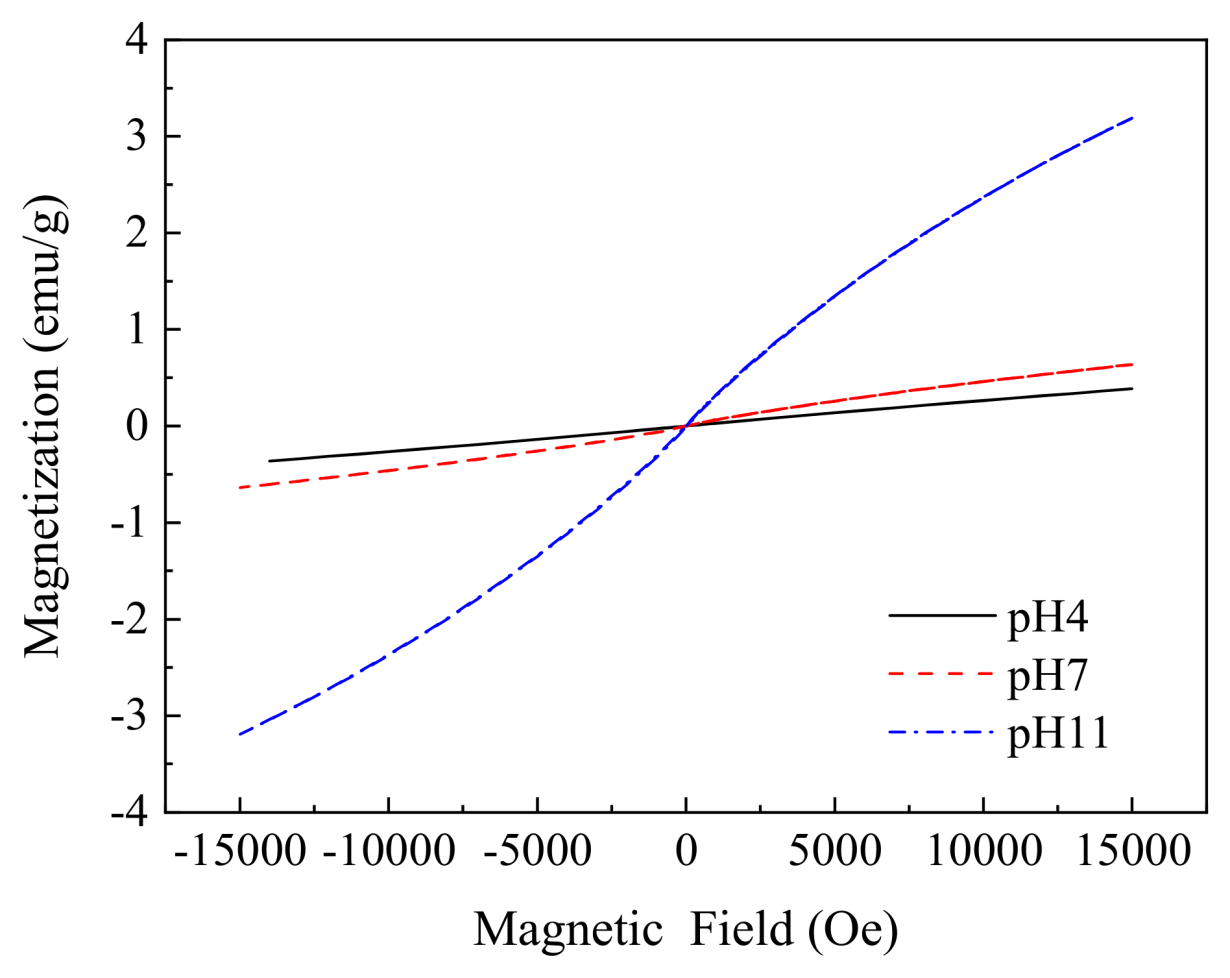
Magnetic hysteresis curves of MAC-4, MAC-7, and MAC-11 measured at 298 K over an applied magnetic field range of −15 to +15 kOe.

The magnetic properties of the prepared materials were evaluated using SQUID magnetometry at 298 K over an applied magnetic-field range of −15 to +15 kOe (Figure 4). The magnetization values measured at approximately 15 kOe were 0.386, 0.638, and 3.190 emu g^−1^ for MAC-4, MAC-7, and MAC-11, respectively. Among the materials prepared at different coprecipitation pH values, MAC-11 exhibited the strongest magnetic response. Because the magnetization curves did not clearly reach saturation within the investigated field range, these values are reported as magnetization at 15 kOe rather than saturation magnetization (M_s_). The magnetic separability of MAC-11 was further demonstrated by dispersing the material in water and placing an external permanent magnet adjacent to the suspension. The particles visibly migrated toward and accumulated near the magnet (Figure S3), demonstrating their responsiveness to an external magnetic field and the feasibility of magnet-assisted separation under the tested conditions. Taken together, the XPS, SQUID, and magnetic-separation results support the incorporation of Fe-containing species and the development of magnetic functionality in MAC-11 without requiring assignment of a specific iron-oxide crystalline phase.

**Figure 5:**
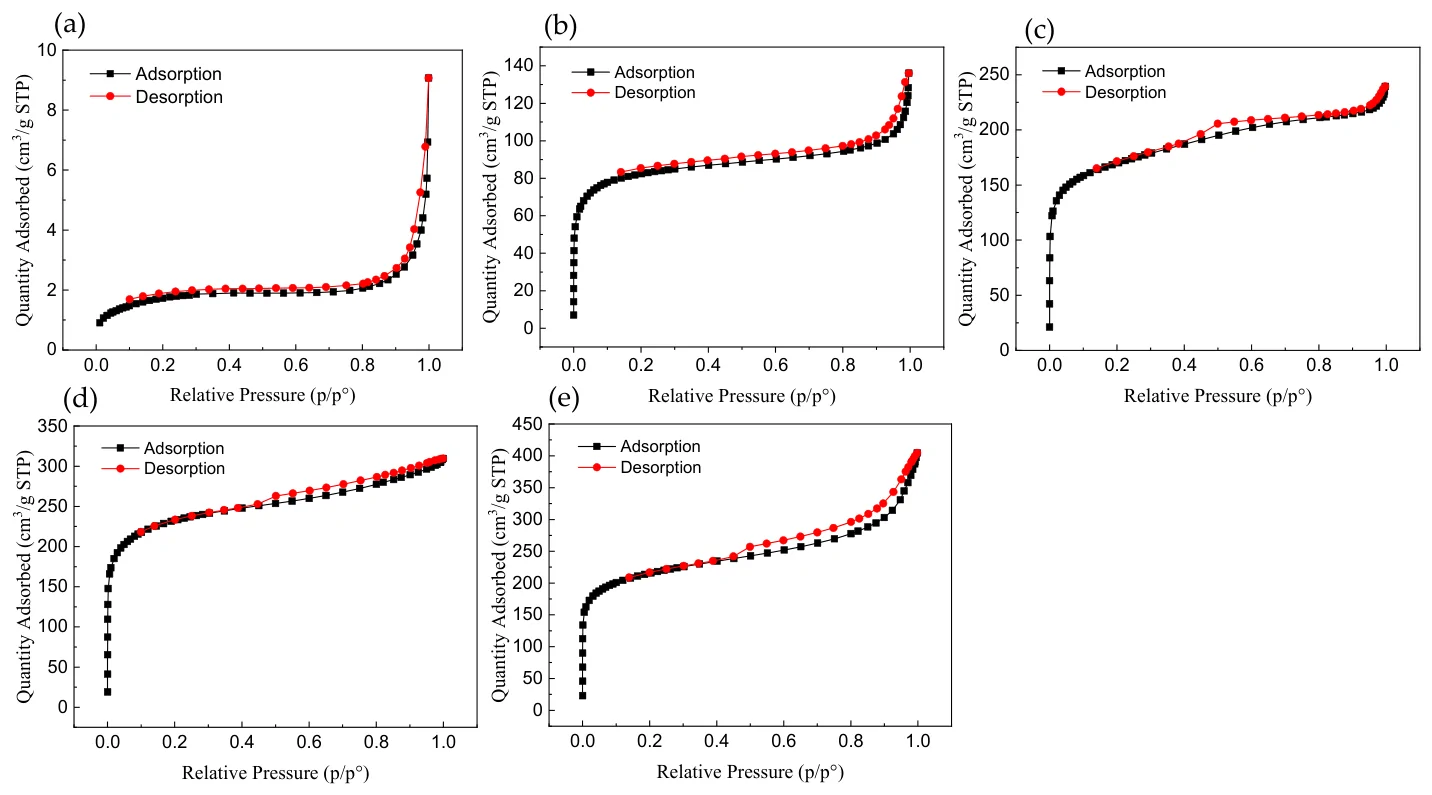
Nitrogen adsorption–desorption isotherms of (**a**) HTC-CB, (**b**) AC-300, (**c**) MAC-11, (**d**) CAC-1, and (**e**) CAC-2.

The textural characteristics of HTC-CB, AC-300, MAC-11, and the commercial activated carbons were evaluated by N_2_ adsorption–desorption analysis (Figure 5), with the corresponding parameters summarized in Table 2. HTC-CB exhibited a BET specific surface area of only 5.96 m^2^ g^−1^ and a total pore volume of 0.00845 cm^3^ g^−1^. Following H_3_PO_4_ activation at 300 °C, these values increased substantially to 312.47 m^2^ g^−1^ and 0.07207 cm^3^ g^−1^, respectively, demonstrating the development of N_2_-accessible porosity under the selected activation conditions. Subsequent magnetic modification further increased the BET specific surface area to 542.41 m^2^ g^−1^ and the total pore volume to 0.19762 cm^3^ g^−1^. For comparison, CAC-1 and CAC-2 exhibited BET specific surface areas of 747.05 and 686.36 m^2^ g^−1^, respectively. The reported average pore sizes were 15.8, 5.34, and 3.84 nm for HTC-CB, AC-300, and MAC-11, respectively, compared with 4.02 and 6.17 nm for CAC-1 and CAC-2.

The BJH desorption pore-size distributions of AC-300 and MAC-11 are compared in Figure S4. MAC-11 exhibited higher differential pore-volume values than AC-300 in the small-mesopore region, particularly at apparent pore diameters of approximately 2–4 nm. This observation is consistent with the higher BJH desorption cumulative pore volume reported for MAC-11 (Table 2). However, pore-size distributions derived from the BJH desorption method should be interpreted cautiously, particularly in this diameter range, because pore-network effects and tensile strength effects can influence the apparent distribution. Accordingly, the observed differences indicate changes in the N_2_-accessible textural characteristics following magnetic modification but do not conclusively establish the formation or blockage of pores within a specific size range.

Notably, the increase in BET specific surface area following magnetic modification was not accompanied by improved DEP removal. Although MAC-11 exhibited a substantially higher BET specific surface area than AC-300 (542.41 versus 312.47 m^2^ g^−1^), its DEP removal efficiency was lower under the corresponding screening conditions. This finding indicates that BET specific surface area alone is insufficient to explain the observed adsorption performance. Magnetic modification also introduced Fe-containing species and altered the measured pore characteristics, potentially affecting the accessibility and chemical environment of adsorption sites. However, the relative contributions of these factors cannot be distinguished from the available data. Therefore, DEP adsorption performance should be considered in relation to both accessible pore structure and surface chemical characteristics rather than total BET specific surface area alone.

Overall, H_3_PO_4_ activation at 300 °C increased the BET specific surface area of HTC-CB from 5.96 to 312.47 m^2^ g^−1^, while subsequent magnetic modification introduced Fe-containing species and imparted sufficient magnetic responsiveness to permit qualitative magnet-assisted separation from water. These results demonstrate the development of N_2_-accessible porosity and magnetic functionality, providing the material characteristics needed for subsequent DEP adsorption experiments.

### 2.3. Effects of Operating Conditions on DEP Adsorption

**Figure 6:**
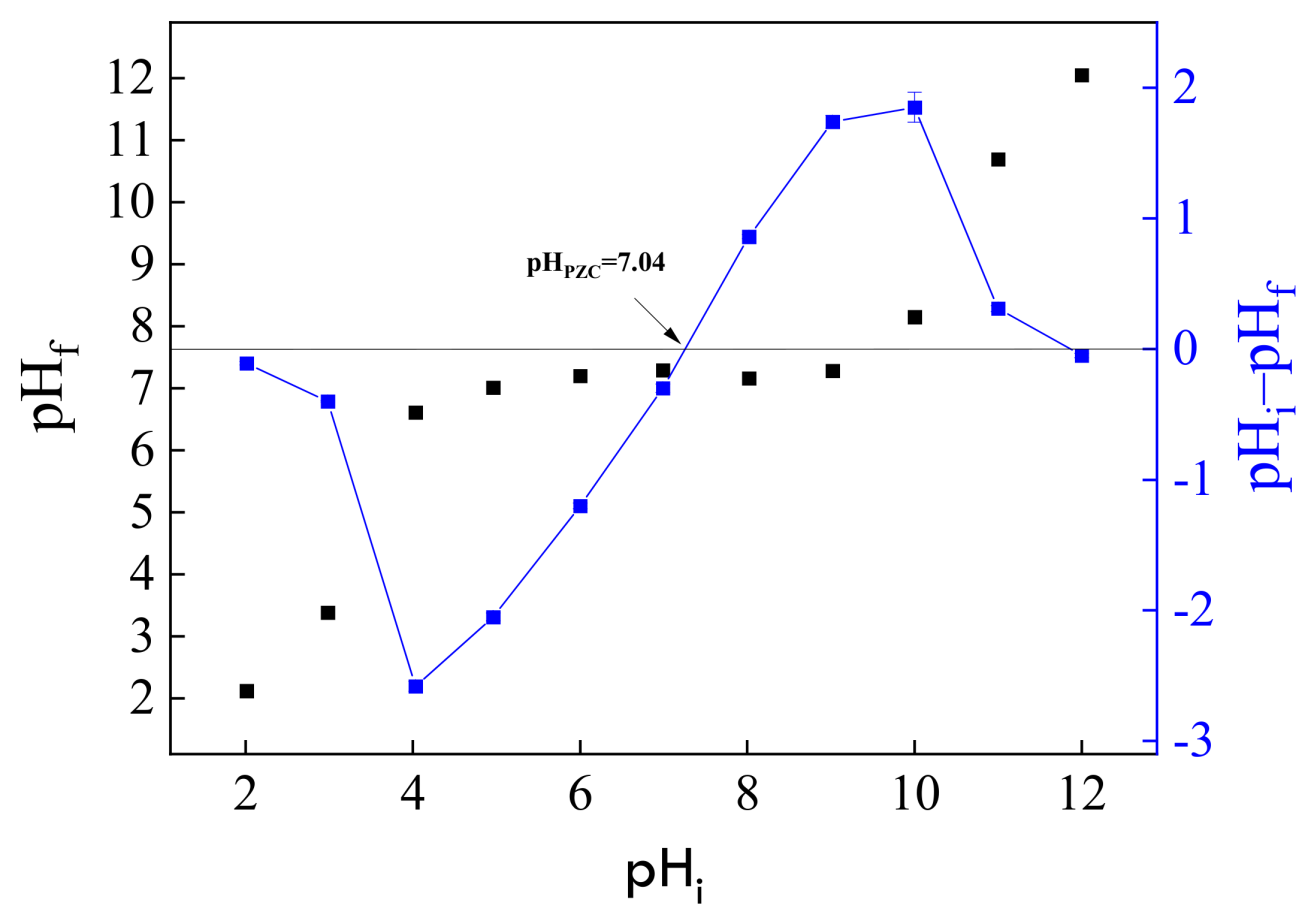
Determination of the point of zero charge (pH_p_zc = 7.04) of MAC-11 using the pH-drift method.

The effects of initial DEP concentration, solution pH, adsorbent dosage, and temperature on DEP removal by MAC-11 were investigated individually (Figure S5). DEP removal decreased progressively as the initial DEP concentration increased from 20 to 60 mg L^−1^ (Figure S5a). At a fixed adsorbent dosage, increasing the initial DEP concentration increases the amount of solute relative to the available adsorption sites, potentially reducing the fraction removed as the adsorption capacity becomes progressively utilized. Solution pH substantially influenced the measured DEP removal efficiency (Figure S5b). At an initial DEP concentration of 20 mg L^−1^, an adsorbent dosage of 10 mg, and a temperature of 30 °C, the highest removal efficiency (98.32%) was observed at pH 3, compared with approximately 76% at pH 2. Between pH 4 and 10, removal efficiencies remained relatively stable at approximately 80–91%, whereas a pronounced decline occurred under strongly alkaline conditions, reaching 19.67% at pH 12. The pH-drift method yielded a point of zero charge (pH_p_zc) of approximately 7.04 for MAC-11 (Figure 6). Because DEP is predominantly present as a neutral molecule in aqueous solution, the observed pH dependence cannot be explained solely by electrostatic attraction or repulsion. Changes in the protonation state and hydration of oxygen- and Fe-containing surface groups may influence their interactions with DEP, potentially affecting hydrogen bonding and other interfacial interactions. Nevertheless, these mechanisms were not directly investigated. Furthermore, because DEP stability and possible non-adsorptive losses were not independently evaluated across the investigated pH range, particularly under strongly acidic and alkaline conditions, the observed changes in removal efficiency cannot be attributed exclusively to adsorption-site chemistry.

The effect of adsorbent dosage was investigated over the range of 10–30 mg (Figure S5c). Increasing the MAC-11 dosage from 10 to 15 mg slightly increased DEP removal from approximately 88% to 89%. However, further increases in dosage were accompanied by progressively lower removal efficiencies, reaching approximately 76% at 30 mg. Although increasing adsorbent dosage generally provides more adsorption sites, a decrease in removal efficiency at higher dosages has also been reported in previous adsorption studies [26]. Particle aggregation or reduced dispersion has been proposed as a possible explanation for such behavior, as these effects may reduce the accessibility of adsorption sites and the effective contact between adsorbent particles and dissolved pollutants [26]. In the present system, dosage-dependent changes in solution pH could also contribute to the observed trend, given the pronounced influence of pH on DEP removal. However, neither particle aggregation nor changes in solution pH with increasing MAC-11 dosage were independently evaluated. Therefore, the mechanism responsible for the observed decrease in removal efficiency at higher adsorbent dosages remains unresolved. Temperature also affected DEP removal by MAC-11 (Figure S5d). Removal efficiency decreased progressively as the temperature increased from 30 to 60 °C, indicating that DEP removal was favored at the lower temperatures examined. However, because adsorption thermodynamic parameters were not determined, the temperature dependence alone is insufficient to establish the thermodynamic nature of the adsorption process.

Overall, the single-factor experiments demonstrated that DEP removal by MAC-11 depended on initial DEP concentration, solution pH, adsorbent dosage, and temperature. These results informed the selection of operating conditions for subsequent experiments. Temperature, adsorbent dosage, and contact time were then investigated simultaneously using a central composite design (CCD) within the RSM framework to evaluate their individual, nonlinear, and interaction effects on DEP removal.

### 2.4. Response Surface Analysis and Optimization

A central composite design (CCD) combined with response surface methodology (RSM) was employed to investigate the combined effects of temperature (X_1_), adsorbent dosage (X_2_), and contact time (X_3_) on DEP removal by MAC-11. The solution volume, initial DEP concentration, and initial solution pH were maintained at 20 mL, 20 mg L^−1^, and 5.8, respectively. The experimental design comprised 20 runs, including six replicated center points (Table S1). Among the candidate regression models, the quadratic model provided the most favorable combination of adjusted R^2^ (0.9539) and predicted R^2^ (0.8301), whereas the cubic model was aliased (Table S2). The quadratic model was therefore selected for subsequent analysis.

Analysis of variance (ANOVA) indicated that the quadratic model was statistically significant (F = 44.69, *p* < 0.0001), with R^2^ = 0.9757, adjusted R^2^ = 0.9539, and a coefficient of variation of 1.46% (Table 3). All three linear terms were significant, with *p* = 0.0123 for temperature (X_1_) and *p* < 0.0001 for both adsorbent dosage (X_2_) and contact time (X_3_). Among the interaction terms, only the temperature–contact time interaction (X_1_X_3_) was significant (F = 21.79, *p* = 0.0009), whereas X_1_X_2_ and X_2_X_3_ were not significant. All three quadratic terms were also significant, with the particularly large F-value for X_3_^2^ (115.36) indicating pronounced curvature in the contact-time response. Based on the F-values of the linear terms, contact time (131.26) and adsorbent dosage (110.51) exhibited stronger statistical effects than temperature (9.30) within the investigated factor ranges. However, these comparisons are specific to the selected experimental domain and do not establish the intrinsic importance of the variables beyond these conditions. The significant X_1_X_3_ interaction further indicates that the effect of temperature depended on contact time, supporting the use of a multivariable experimental design.

**Figure 7:**
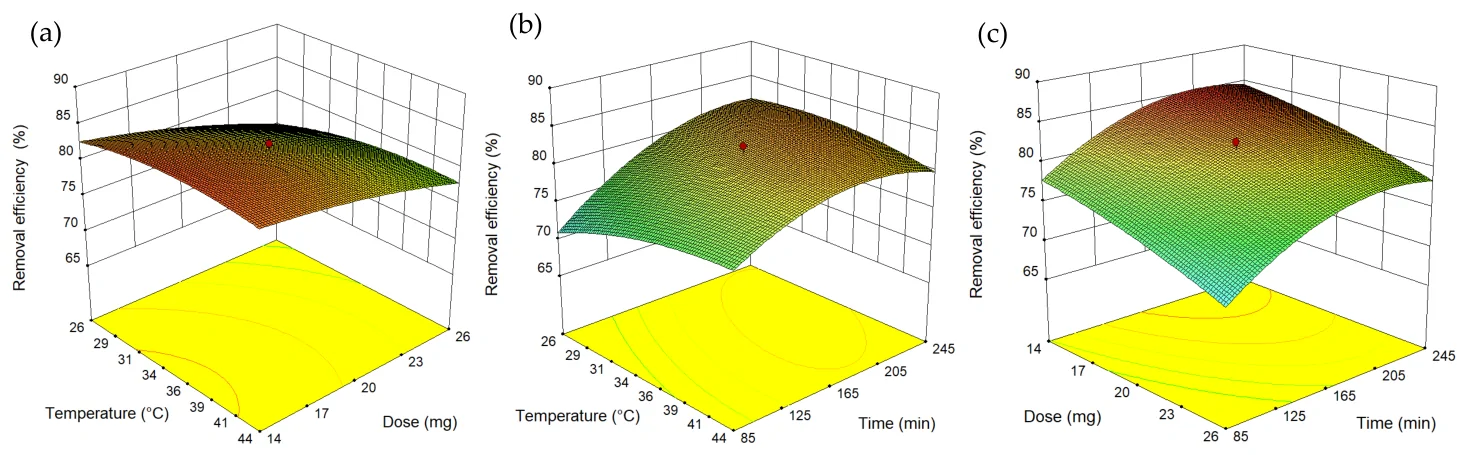
Response surface plots showing the combined effects of operating variables on DEP removal by MAC-11: (**a**) temperature and adsorbent dosage, (**b**) temperature and contact time, and (**c**) adsorbent dosage and contact time.

The three-dimensional response surfaces are presented in Figure 7. The temperature–dosage and dosage–time surfaces exhibited relatively weak interaction patterns, consistent with the nonsignificant X_1_X_2_ and X_2_X_3_ terms. In contrast, the temperature–time surface showed a more pronounced interaction, consistent with the significant X_1_X_3_ term. The curvature of the surfaces was also consistent with the significant quadratic terms. The six replicated center-point experiments conducted at 35 °C, 20 mg adsorbent, and 165 min exhibited relatively small experimental variability, with a pure-error sum of squares of 1.39 (Table 3). Nevertheless, the lack-of-fit test was statistically significant (F = 8.42, *p* = 0.0177), indicating that the quadratic model did not fully account for the systematic variation in DEP removal relative to the experimental pure error.

Additional diagnostic plots were examined to assess the residual behavior (Figure S6). Most externally studentized residuals were distributed approximately along the reference line in the normal probability plot, although deviations were observed at the tails. The residuals-versus-predicted and residuals-versus-run plots showed no pronounced funnel-shaped pattern or systematic trend. Two observations exhibited relatively large externally studentized residuals; however, no documented experimental errors justified their exclusion, and all 20 runs were retained. These diagnostic results did not eliminate the concern raised by the statistically significant lack of fit. Consequently, despite the relatively high R^2^, the quadratic model should be regarded as an empirical approximation with limitations in predictive accuracy, including within the investigated experimental domain.

Given these limitations, the quadratic model was used primarily to identify a promising operating condition within the investigated factor ranges rather than to establish a definitive global optimum. Numerical optimization identified a candidate condition of 30.4 °C, 14.6 mg MAC-11, and 236 min, with a predicted DEP removal efficiency of 85.0%. An independent confirmation experiment conducted under these conditions yielded a removal efficiency of 87.7 ± 0.37%, corresponding to a difference of 2.7 percentage points from the predicted value. Although this result does not establish the predictive accuracy of the model throughout the experimental domain, it demonstrates that the model-selected condition provided substantial DEP removal in the confirmation experiment. Accordingly, this condition was adopted as an experimentally confirmed operating condition for the subsequent adsorption kinetic and equilibrium studies.

### 2.5. Adsorption Kinetics and Equilibrium Behavior

**Figure 8:**
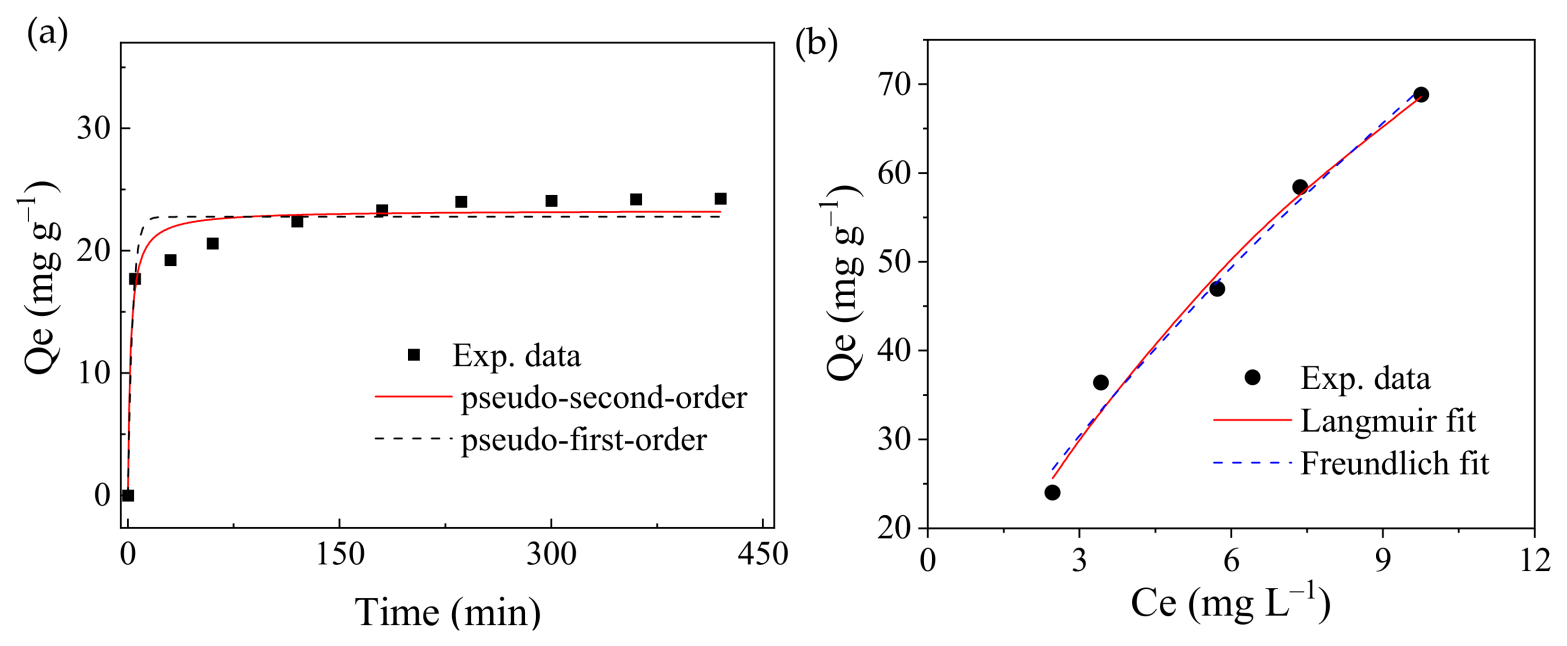
Kinetic and equilibrium modeling of DEP adsorption onto MAC-11: (**a**) experimental kinetic data fitted using the nonlinear pseudo-first-order (PFO) and pseudo-second-order (PSO) models; (**b**) experimental equilibrium data fitted using the nonlinear Langmuir and Freundlich isotherm models at 30.4 °C.

DEP adsorption kinetics on MAC-11 were investigated at the RSM-selected temperature (30.4 °C) and adsorbent dosage (14.6 mg), using an initial DEP concentration of 20 mg L^−1^ and an initial solution pH of 5.8. The adsorption experiments were conducted for up to 420 min. DEP uptake was relatively rapid during the initial period, followed by a gradual decrease in the uptake rate as the contact time increased (Figure 8a). The experimentally determined adsorption capacity at the final sampling time was 24.3 mg g^−1^ (Table 4). Nonlinear fitting indicated that the pseudo-second-order (PSO) model provided a slightly better empirical description of the kinetic data (R^2^ = 0.9633) than the pseudo-first-order (PFO) model (R^2^ = 0.9422). The adsorption capacity estimated by the PSO model (23.3 mg g^−1^) was also closer to the experimentally measured value (24.3 mg g^−1^) than that estimated by the PFO model (22.8 mg g^−1^). The corresponding rate constants were 0.022 g mg^−1^ min^−1^ for PSO and 0.3002 min^−1^ for PFO (Table 4). However, the better PSO fit should be interpreted as an empirical description of the uptake profile rather than direct evidence of a chemisorption mechanism.

To further examine the mass-transfer behavior, the kinetic data were analyzed using the Weber–Morris intraparticle diffusion and Boyd models (Figure S7). The Weber–Morris plot exhibited three apparent uptake regions. The initial region (5–60 min) had a fitted slope of 0.5199 mg g^−1^ min^−1^/^2^, followed by a lower slope of 0.3675 mg g^−1^ min^−1^/^2^ over 120–236 min. In the final region (300–420 min), the slope decreased to 0.0503 mg g^−1^ min^−1^/^2^, consistent with a progressively slower uptake rate. The positive intercepts of the fitted segments suggest that intraparticle diffusion was not the sole process influencing the adsorption kinetics. Nevertheless, these apparent regions should not be interpreted as definitive evidence of separate rate-controlling mechanisms.

The Boyd plot showed an approximately linear relationship between the transformed uptake parameter and contact time, described by B_t_ = 0.01274t + 0.6435 (R^2^ = 0.9898). Because the fitted line did not pass through the origin, the results suggest that intraparticle diffusion alone may not adequately describe the overall adsorption kinetics. External mass transfer or other early-stage processes may also contribute. However, the first measurement was obtained at 5 min, and the Boyd transformation is sensitive to the estimated equilibrium uptake, particularly as the fractional uptake approaches unity. Therefore, the available data do not permit an unambiguous identification of the rate-limiting step. Collectively, the diffusion-model analyses suggest that DEP uptake involves contributions from more than one mass-transfer process.

Equilibrium adsorption behavior was investigated using initial DEP concentrations of 20–60 mg L^−1^ and the RSM-selected contact time of 236 min. The resulting adsorption data were fitted to the nonlinear Langmuir and Freundlich isotherm models (Figure 8b). Both models provided similarly high coefficients of determination, with R^2^ values of 0.9834 and 0.9805, respectively. The small difference in goodness of fit does not provide sufficient evidence to identify either model as a unique description of the adsorption mechanism.

The Langmuir model yielded an estimated maximum adsorption capacity (*q*_max_) of 158.5 mg g^−1^. However, the experimental concentration range did not establish a clear saturation plateau. Accordingly, this value represents an extrapolated model parameter rather than an experimentally demonstrated maximum adsorption capacity. Previous research has also highlighted the sensitivity of Langmuir-derived maximum capacities to the concentration range used for model fitting, particularly when saturation is not experimentally observed [27]. The concentration range used in the present study was selected to evaluate DEP adsorption under controlled aqueous conditions rather than to determine the ultimate saturation capacity. Because the investigated concentrations were substantially higher than those typically encountered in municipal wastewater, these findings should not be directly extrapolated to DEP removal at environmentally relevant trace concentrations. The estimated *q*_max_ is therefore reported for model comparison purposes only, and comparisons with other adsorbents should emphasize experimentally measured adsorption capacities and comparable operating conditions. Additional equilibrium experiments extending to higher surface coverage would be required to establish the actual saturation capacity of MAC-11.

Overall, the kinetic results showed relatively rapid initial DEP uptake followed by progressively slower adsorption, with the PSO model providing the better empirical fit. Both the Langmuir and Freundlich models described the concentration-dependent adsorption data, although neither model established a unique adsorption mechanism or experimentally verified saturation capacity. These findings demonstrate that MAC-11 retained measurable DEP adsorption capability following magnetic modification.

### 2.6. Comparison of Adsorption Performance

The DEP adsorption performances of AC-300, MAC-11, and two commercial activated carbons were compared under identical experimental conditions (Table S3). AC-300 achieved a DEP removal efficiency of 98.6% and an adsorption capacity of 27.00 mg g^−1^, whereas MAC-11 exhibited 87.0% removal and an adsorption capacity of 23.85 mg g^−1^. The commercial activated carbons, CAC-1 and CAC-2, achieved removal efficiencies of 99.6% and 99.3%, with corresponding adsorption capacities of 27.29 and 27.21 mg g^−1^, respectively. These results demonstrate that magnetic modification reduced DEP uptake relative to AC-300 under the investigated conditions, although MAC-11 retained substantial adsorption performance while acquiring magnetic functionality. Importantly, the adsorption capacities in Table S3 were obtained under a single set of experimental conditions and should not be interpreted as maximum adsorption capacities. In particular, the near-complete DEP removal achieved by AC-300 and the commercial activated carbons indicates that their measured capacities were largely constrained by the amount of DEP initially available rather than by saturation of their adsorption sites. Therefore, these results provide a controlled comparison of DEP removal performance but do not establish the relative maximum adsorption capacities of the materials.

The decrease in DEP removal following magnetic modification occurred despite an increase in BET specific surface area from 312.47 m^2^ g^−1^ for AC-300 to 542.41 m^2^ g^−1^ for MAC-11, accompanied by an increase in total pore volume from 0.07207 to 0.19762 cm^3^ g^−1^ (Table 2). The reported average pore size decreased from 5.34 to 3.84 nm, while the BJH desorption pore-size distributions showed higher apparent differential pore volumes for MAC-11 in the small-mesopore region (Figure S4). Although BJH desorption results require cautious interpretation because of possible pore-network and tensile strength effects, the measured textural properties do not provide direct evidence that extensive pore blockage was responsible for the reduced DEP removal. Previous studies have reported that iron-oxide incorporation can influence pore accessibility and adsorption behavior in magnetic activated carbons [28]. However, the extent to which such effects occurred in the present materials cannot be established from the available measurements.

Changes in surface chemistry may also contribute to differences in DEP adsorption following magnetic modification. Han et al. [29] reported that magnetite impregnation affected the adsorption of organic compounds differently depending on their physicochemical properties, illustrating that magnetic modification does not necessarily produce a uniform response across adsorbates. In the present study, Fe-containing species introduced during coprecipitation may have altered the accessibility and chemical environment of carbon adsorption sites. Potential DEP adsorption interactions include hydrogen bonding between surface oxygen-containing groups and DEP ester groups, π–π interactions involving aromatic carbon domains and the DEP aromatic ring, and adsorption within accessible pores. Similar contributions from pore filling, hydrogen bonding, hydrophobic interactions, and π–π interactions have been proposed for phthalate adsorption onto biochars and related carbonaceous materials [30,31]. Nevertheless, the FTIR and XPS measurements in the present study were performed before adsorption and do not directly identify the interactions responsible for DEP uptake. These mechanisms should therefore be regarded as plausible explanations rather than experimentally confirmed adsorption pathways.

The principal advantage of MAC-11 is the combination of a waste-derived cellulose-acetate precursor, H_3_PO_4_ activation at a comparatively low temperature of 300 °C, measurable DEP adsorption performance, and magnetic separability. The SQUID measurements and external-magnet separation test demonstrated that MAC-11 responded to an applied magnetic field and could be separated qualitatively from an aqueous suspension. However, magnetic recoverability should not be equated with regenerability or reusability. DEP desorption, repeated adsorption–desorption cycles, and Fe leaching were not investigated in the present study. Consequently, the retention of adsorption capacity and magnetic functionality after regeneration remains unknown. Further evaluation of regeneration behavior, Fe release, and adsorption performance in complex water matrices is necessary before the long-term applicability of MAC-11 can be established.

Comparison with previously reported DEP adsorbents also requires consideration of differences in experimental conditions, particularly initial pollutant concentration, adsorbent dosage, contact time, and the extent to which adsorption equilibrium and saturation were established. Previous methodological studies have emphasized the limitations of comparing adsorbents solely on the basis of removal efficiency or regression-derived maximum adsorption capacity [27,32]. As discussed in Section 3.5, the Langmuir-derived maximum adsorption capacity of MAC-11 (158.5 mg g^−1^) was estimated from a limited concentration range without an experimentally observed saturation plateau. It should therefore be treated as an extrapolated model parameter rather than a verified maximum capacity. The comparison in Table S3 provides a more direct assessment of relative DEP removal performance under identical conditions, although it does not establish the intrinsic adsorption capacities of the materials.

The environmental implications of converting discarded cigarette filters into adsorbents also warrant consideration. Previous studies have shown that hydrothermal carbonization can alter the distribution and chemical forms of contaminants, including heavy metals and organic compounds, between solid and aqueous phases [33,34,35]. Hydrothermal process water may also contain dissolved organic and inorganic constituents requiring appropriate management [36]. In addition, thermochemical conversion of discarded cigarette butts has been reported to generate various organic products under pyrolysis conditions [37]. However, these findings cannot be directly extrapolated to the present process, which involved hydrothermal carbonization at 180 °C followed by H_3_PO_4_ activation at 300 °C. Although ethanol washing and thermal treatment may reduce or transform certain contaminants, their complete removal or destruction has not been demonstrated. Contaminant-specific analyses of the resulting solids and process liquids, together with leaching assessments of MAC-11, are therefore needed to establish the environmental safety of the prepared adsorbent.

## 3. Materials and Methods

### 3.1. Chemicals and Materials

Discarded cigarette filters were collected from dedicated collection boxes installed at major cigarette-littering hotspots in Taipei City, Taiwan, as part of a routine recycling program. Two commercially available activated carbons, supplied by Emperor Chemical Co., Ltd. (Taipei, Taiwan) and Microtest Corporation (New Taipei City, Taiwan), were used as reference adsorbents and designated CAC-1 and CAC-2, respectively. Diethyl phthalate (DEP; C_12_H_14_O_4_; CAS No. 84-66-2), iron(III) chloride hexahydrate (FeCl_3_·6H_2_O, ≥99%), and iron(II) chloride tetrahydrate (FeCl_2_·4H_2_O, ≥99%) were purchased from Acros Organics (Geel, Belgium). Phosphoric acid (H_3_PO_4_, 85%) and sodium hydroxide (NaOH) were obtained from Fisher Chemical (Waltham, MA, USA), whereas hydrochloric acid (HCl) was supplied by Scharlau (Barcelona, Spain). All chemicals were of analytical grade and used as received without further purification.

### 3.2. Pretreatment of Discarded Cigarette Filters

The wrapping paper was manually removed from the collected cigarette butts, and only the filter portions were retained for subsequent processing. The separated filters were immersed in ethanol for 24 h, followed by vacuum filtration and repeated ethanol washing until their color changed from yellowish-brown to off-white. The washed filters were then oven-dried before carbon preparation. To assess the effectiveness of the pretreatment procedure, samples of untreated and ethanol-washed filters were analyzed using UV–Vis spectroscopy and inductively coupled plasma optical emission spectrometry (ICP-OES) to evaluate changes in UV-absorbing constituents and selected metal contents, respectively.

### 3.3. Preparation of Cigarette-Filter-Derived Porous Carbons

#### 3.3.1. Hydrothermal Carbonization

The pretreated cigarette filters were subjected to hydrothermal carbonization (HTC) to produce hydrochar. Briefly, 10 g of dried cigarette filters and 30 mL of deionized water were loaded into an autoclave. The reactor was heated to 180 °C at a rate of 10 °C min^−1^ and maintained at this temperature for 24 h. After cooling to room temperature, the resulting solid was recovered by vacuum filtration, washed repeatedly with deionized water, and oven-dried. The dried product was subsequently ground using a mortar and stored in a sealed container at room temperature. The resulting hydrochar was designated HTC-CB.

#### 3.3.2. H_3_PO_4_ Activation

For each activation batch, 1.0 g of dried HTC-CB was thoroughly mixed with an aqueous H_3_PO_4_ solution at the specified concentration and mass ratio. The mixture was then thermally activated in a quartz tube under a continuous N_2_ flow of 10 mL min^−1^. To investigate the effect of activation temperature, the furnace was heated at 5 °C min^−1^ to 300, 400, 500, 600, 700, or 800 °C and held at the target temperature for 2 h. After cooling to room temperature under N_2_, the activated products were recovered, repeatedly washed with deionized water by vacuum filtration, dried, ground, and stored in sealed containers. The resulting activated carbons were designated AC-300, AC-400, AC-500, AC-600, AC-700, and AC-800 according to their respective activation temperatures.

Following the activation-temperature screening, the effects of H_3_PO_4_ concentration and impregnation ratio were investigated at the selected activation temperature of 300 °C. The influence of acid concentration was first examined using 10, 40, and 85 wt% H_3_PO_4_ solutions. Subsequently, the mass ratio of dried HTC-CB to H_3_PO_4_ solution was varied from 1:2 to 1:6 (1:2, 1:3, 1:4, 1:5, and 1:6) using an 85 wt% H_3_PO_4_ solution. For clarity, a mass ratio of 1:3 corresponded to mixing 1.0 g of dried HTC-CB with 3.0 g of the H_3_PO_4_ solution, rather than 3.0 g of pure H_3_PO_4_. All experiments were conducted under the same N_2_ flow rate (10 mL min^−1^), heating rate (5 °C min^−1^), and holding time (2 h), with the activation temperature maintained at 300 °C for the subsequent concentration and impregnation-ratio experiments.

#### 3.3.3. Magnetic Modification

Magnetic modification of the selected activated carbon was carried out via Fe(II)/Fe(III) coprecipitation. Briefly, 2.0 g of activated carbon, 2.7 g of FeCl_3_·6H_2_O, and 1.2 g of FeCl_2_·4H_2_O were dispersed in 100 mL of deionized water. The pH of the suspension was adjusted to 4, 7, or 11 using 1 M NaOH, and the mixture was subsequently maintained in a thermostatically controlled shaking water bath at 85 °C and 100 rpm for 1 h. The resulting solids were recovered by vacuum filtration and washed repeatedly with deionized water until the filtrate reached approximately neutral pH. The washed products were dried overnight at 50 °C, ground, passed through a 100-mesh sieve, and stored in sealed containers at room temperature. The materials prepared at initial suspension pH values of 4, 7, and 11 were designated MAC-4, MAC-7, and MAC-11, respectively. Based on the preparation-condition screening results presented in Section 3.1, MAC-11 was selected for further physicochemical characterization and DEP adsorption experiments.

### 3.4. Characterization

The surface morphology of the prepared materials was examined by scanning electron microscopy (SEM; S3000H, Hitachi, Tokyo, Japan) at an accelerating voltage of 15 kV after gold coating for 90 s. Surface functional groups were characterized by Fourier-transform infrared spectroscopy (FTIR; Spectrum 100, PerkinElmer, Waltham, MA, USA), while surface elemental composition and chemical states were investigated by X-ray photoelectron spectroscopy (XPS; Quantes, ULVAC-PHI, Chigasaki, Japan). Crystalline structures were examined by X-ray diffraction (XRD; D8 SSS, Bruker, Billerica, MA, USA). N_2_ adsorption–desorption isotherms were measured using a surface area and porosity analyzer (TriStar II Plus, Micromeritics, Norcross, GA, USA) to evaluate the Brunauer–Emmett–Teller (BET) specific surface area, pore volume, and pore-size characteristics. Bulk elemental composition was determined using an elemental analyzer (vario EL cube, Elementar, Langenselbold, Germany). Magnetic properties were evaluated using a superconducting quantum interference device (SQUID) magnetometer (MPMS 3, Quantum Design, San Diego, CA, USA) at 298 K over an applied magnetic-field range of −15 to +15 kOe. Magnetic separability was further assessed qualitatively by dispersing MAC-11 in water and placing an external permanent magnet adjacent to the suspension. The migration and accumulation of particles toward the magnet were visually observed and recorded.

### 3.5. DEP Adsorption Experiments

The DEP adsorption performance of the prepared carbon materials was evaluated through batch adsorption experiments. DEP concentrations were quantified using a UV–Vis spectrophotometer (V-700, JASCO, Tokyo, Japan) at 231 nm, corresponding to the maximum absorbance observed in the DEP absorption spectrum, with concentrations determined from an external calibration curve. To screen the carbon preparation conditions, 10 mg of adsorbent was added to 20 mL of an aqueous DEP solution (100 mg L^−1^), and the suspension was shaken at 30 °C and 100 rpm for 24 h. Following adsorption, the suspension was centrifuged at 6000 rpm for 10 min, and the residual DEP concentration in the supernatant was determined by UV–Vis spectroscopy. This procedure was applied to assess the effects of activation temperature, H_3_PO_4_ concentration, HTC-CB-to-H_3_PO_4_ solution mass ratio, and pH during magnetic modification. All preparation-condition screening experiments were performed in triplicate, and the results are reported as mean ± standard deviation.

Following the selection of MAC-11, the effects of initial DEP concentration, solution pH, adsorbent dosage, and temperature on DEP removal were investigated individually. Unless otherwise specified, experiments were performed using 20 mL of DEP solution at a shaking speed of 100 rpm for 24 h. Initial DEP concentrations ranged from 20 to 60 mg L^−1^, while adsorbent dosage and temperature were varied from 10 to 30 mg and from 30 to 60 °C, respectively. After adsorption, the suspensions were centrifuged, and the residual DEP concentrations in the supernatants were quantified by UV–Vis spectroscopy. DEP removal efficiency and adsorption capacity were calculated from the initial and residual DEP concentrations using conventional mass-balance relationships.

### 3.6. Experimental Design and Statistical Analysis

Response surface methodology (RSM) based on a central composite design (CCD) was employed to investigate the individual and interactive effects of temperature (X_1_), adsorbent dosage (X_2_), and contact time (X_3_) on DEP removal efficiency using MAC-11. The experimental design and statistical analyses were performed using Design-Expert software (version 13). The investigated ranges were 20–50 °C for temperature, 10–30 mg for adsorbent dosage, and 30–300 min for contact time. The CCD consisted of 20 experimental runs, including factorial points, axial points, and six replicated center points. Linear, two-factor interaction (2FI), quadratic, and cubic models were evaluated, and a quadratic model was selected based on the model-fitting statistics. Analysis of variance (ANOVA) was performed to assess the statistical significance of the selected model and its individual terms. The complete experimental design matrix and corresponding coded factor levels are presented in Table S1, while the model-selection statistics are summarized in Table S2. Model adequacy was further examined using normal probability plots, residuals-versus-predicted and residuals-versus-run plots, externally studentized residuals, and the lack-of-fit test. These diagnostic analyses were used to assess the statistical behavior and limitations of the fitted model before its application to response prediction and optimization within the investigated experimental domain.

### 3.7. Adsorption Kinetics and Equilibrium Studies

Adsorption kinetic experiments were conducted using MAC-11 at the optimal temperature identified by response surface methodology (RSM). Briefly, MAC-11 was added to 20 mL of an aqueous DEP solution (20 mg L^−1^), and the suspension was shaken at 100 rpm for contact times of up to 7 h. At predetermined contact times, the suspensions were centrifuged at 6000 rpm for 10 min, and the residual DEP concentrations in the supernatants were quantified by UV–Vis spectroscopy. The experimental kinetic data were fitted to the nonlinear pseudo-first-order (PFO) and pseudo-second-order (PSO) kinetic models [38,39]. To further examine the potential contributions of diffusion processes to DEP uptake, the kinetic data were also analyzed using the Weber–Morris intraparticle diffusion and Boyd diffusion models [40,41].

Equilibrium adsorption experiments were performed using initial DEP concentrations ranging from 20 to 60 mg L^−1^, with the contact time fixed at the optimal value identified by RSM. For each adsorption sample, the DEP concentration in the supernatant was measured three times by UV–Vis spectroscopy, and the mean concentration was used to calculate the adsorption capacity. The resulting data were fitted to the nonlinear Langmuir and Freundlich isotherm models [42,43].

## 4. Conclusions

Discarded cellulose-acetate cigarette filters were converted into magnetic porous carbon through hydrothermal carbonization, H_3_PO_4_ activation at 300 °C, and subsequent magnetic modification. H_3_PO_4_ activation increased the BET specific surface area of the hydrochar from 5.96 to 312.47 m^2^ g^−1^, while magnetic modification further increased it to 542.41 m^2^ g^−1^ and imparted sufficient magnetic responsiveness for separation from an aqueous suspension using an external magnet. These findings demonstrate the feasibility of developing porous, magnetically responsive carbon materials from discarded cigarette filters at a comparatively low activation temperature. DEP removal by the magnetically modified carbon (MAC-11) was influenced by the operating conditions. Response surface methodology identified candidate conditions of 30.4 °C, 14.6 mg adsorbent, and 236 min, under which an independent confirmation experiment achieved 87.7% DEP removal. Although the quadratic model exhibited a high coefficient of determination (R^2^ = 0.9757), its statistically significant lack of fit indicates limitations in predictive accuracy. The adsorption kinetics were better described by the pseudo-second-order model, while both the Langmuir and Freundlich models provided comparable fits to the concentration-dependent adsorption data. However, these empirical models did not establish a unique adsorption mechanism or an experimentally verified maximum adsorption capacity. Magnetic modification reduced DEP adsorption performance relative to nonmagnetic AC-300 despite increasing the BET specific surface area. Nevertheless, MAC-11 retained appreciable DEP adsorption capability while acquiring magnetic separability, highlighting a trade-off between adsorption performance and ease of adsorbent recovery. Overall, this study demonstrates the potential of integrating cigarette-filter waste valorization, comparatively low-temperature porous-carbon preparation, DEP adsorption, and magnetic separation within a single material platform. Further investigations of adsorption–desorption cycling, regeneration efficiency, Fe leaching, contaminant release, and performance in complex water matrices are needed to establish the environmental safety, reusability, and practical applicability of MAC-11.

## Figures and Tables

**Table 1:** Elemental compositions of cigarette-filter-derived and commercial activated carbons.

Samples	N (%)	C (%)	H (%)	S (%)
HTC–CB	0.07	64.61	2.52	0.04
AC-300	0.01	49.34	2.28	0.00
MAC-11	0.01	30.69	2.28	0.00
CAC-1	0.70	80.94	2.83	0.23
CAC-2	0.44	76.09	2.74	0.63

**Table 2:** Textural properties of cigarette-filter-derived and commercial activated carbons.

Samples	Surface Area (m^2^ g^−1^)	Pore Volume (cm^3^ g^−1^)	Pore Size (nm)
HTC–CB	5.96	0.008454	15.8
AC-300	312.47	0.072068	5.34
MAC-11	542.41	0.197616	3.84
CAC-1	747.05	0.194157	4.02
CAC-2	686.36	0.415050	6.17

**Table 3:** Analysis of variance for the quadratic model describing DEP removal by MAC-11.

Source	DF	SS	*F* Value	*p* Value
Model	9	526.07	44.69	<0.0001
X_1_	1	12.16	9.3	0.0123
X_2_	1	144.55	110.51	<0.0001
X_3_	1	171.69	131.26	<0.0001
X_1_ X_2_	1	1.9	1.45	0.2557
X_1_ X_3_	1	28.5	21.79	0.0009
X_2_ X_3_	1	0.2112	0.1615	0.6962
X_1_^2^	1	25.37	19.39	0.0013
X_2_^2^	1	7.59	5.8	0.0368
X_3_^2^	1	150.9	115.36	<0.0001
Residual	10	13.08		
Lack of fit	5	11.69	8.42	0.0177
$R^{2}$ = 0.9757				
Adj $R^{2}$ = 0.9539				
C.V. % = 1.46				

**Table 4:** Kinetic and equilibrium model parameters for DEP adsorption on MAC-11.

Model	Parameter	Value
Experimental	*q_exp_* (mg g^−1^)	24.3
Kinetic models		
Pseudo-first-order (nonlinear)	*k*_1_ (min^−1^)	0.3002
	*q_e_* (mg g^−1^)	22.8
	R^2^	0.9422
Pseudo-second-order (nonlinear)	*k*_2_ (g mg^−1^ min^−1^)	0.022
	*q_e_* (mg g^−1^)	23.3
	R^2^	0.9633
Equilibrium models		
Langmuir (nonlinear)	*q_m_* (mg g^−1^)	158.5
	*K_L_* (L mg^−1^)	0.078
	R^2^	0.9834
Freundlich (nonlinear)	*K_F_* (mg g^−1^)(L mg^−1^)^1/n^	1.433
	*n*	14.19
	R^2^	0.9805

Note: The Langmuir-derived *q_m_* is an extrapolated model parameter obtained from the investigated initial DEP concentration range of 20–60 mg L^−1^. It should not be interpreted as an experimentally verified saturation capacity.

## Data Availability

The data presented in this study are available in this article.

## Supplementary Materials

The following supporting information can be downloaded at: https://www.mdpi.com/article/10.3390/molecules31213979/s1, Table S1: Central composite design matrix with the corresponding coded values and experimental DEP removal efficiencies; Table S2: Sequential model comparison for selection of the response surface model for DEP removal; Table S3: Comparison of DEP adsorption performance of cigarette-filter-derived and commercial activated carbons under identical experimental conditions; Figure S1: Evaluation of the effectiveness of ethanol washing for discarded cigarette filters: (a) ICP-OES results for selected elements before and after cleaning; (b) UV–Vis absorption spectra obtained before and after cleaning; Figure S2: XRD patterns of (a) HTC-CB, (b) AC-300, (c) MAC-11, (d) CAC-1, and (e) CAC-2; Figure S3: Magnetic response and separation of MAC-11: (a) MAC-11 before dispersion, (b) MAC-11 dispersed in water, and (c) collection of MAC-11 using an external magnet; Figure S4: BJH desorption pore-size distributions of AC-300 and MAC-11 obtained from N_2_ adsorption–desorption measurements at 77 K. Differential pore volumes are expressed per logarithmic pore-width interval; Figure S5: Effects of operating conditions on DEP removal by MAC-11: (a) initial DEP concentration, (b) solution pH, (c) adsorbent dosage, and (d) temperature; Figure S6: Residual diagnostic plots for the quadratic response surface model: (a) normal probability plot of externally studentized residuals, (b) externally studentized residuals versus predicted DEP removal efficiency, and (c) externally studentized residuals versus experimental run number; Figure S7: Diffusion-model analysis of DEP adsorption onto MAC-11: (a) Weber–Morris intraparticle diffusion plot showing three apparent uptake regions and (b) Boyd diffusion plot with linear regression.
