# Supplementary material for: Low-Temperature Conversion of Discarded Cigarette Filters into Magnetic Porous Carbon for Diethyl Phthalate Removal
Source: Molecules. 2026 Nov 4;31(21):3979. doi: 10.3390/molecules31213979 (PMC13764916; doi:10.3390/molecules31213979)
Supplement: Supplementary file 1 [file molecules-31-03979-s001.zip › molecules-4668886-supplementary.pdf]

Table S1. Central composite design matrix with the corresponding coded values and experimental DEP removal efficiencies.

Run	Independent variables (coded)			Responses
	X_1: temperature (°C)	X_2: adsorbent dosage (mg)	X_3: time (min)	Y: removal efficiency (%)
1	26.1 (-1)	14.0 (-1)	85 (-1)	73.8
2	44.0 (1)	14.0 (-1)	85 (-1)	79.8
3	26.1 (-1)	26.0 (1)	85 (-1)	66.5
4	44.0 (1)	26.0 (1)	85 (-1)	73.4
5	26.1 (-1)	14.0 (-1)	245 (1)	83.7
6	44.0 (1)	14.0 (-1)	245 (1)	81.1
7	26.1 (-1)	26.0 (1)	245 (1)	76.0
8	44.0 (1)	26.0 (1)	245 (1)	76.4
9	20.0 (-1.68)	20.0 (0)	165 (0)	77.9
10	50.0 (1.68)	20.0 (0)	165 (0)	79.2
11	35.0 (0)	10.0 (-1.68)	165 (0)	85.7
12	35.0 (0)	30.0 (1.68)	165 (0)	74.8
13	35.0 (0)	20.0 (0)	30 (-1.68)	65.8
14	35.0 (0)	20.0 (0)	300 (1.68)	80.5
15	35.0 (0)	20.0 (0)	165 (0)	81.6
16	35.0 (0)	20.0 (0)	165 (0)	82.9
17	35.0 (0)	20.0 (0)	165 (0)	81.8
18	35.0 (0)	20.0 (0)	165 (0)	81.7
19	35.0 (0)	20.0 (0)	165 (0)	81.4
20	35.0 (0)	20.0 (0)	165 (0)	81.9

Table S2. Sequential model comparison for selection of the response surface model for DEP removal.

Source	Sequential p-value	Adjusted R^2	Predicted R^2	
Linear	0.0015	0.5358	0.3867	
2FI	0.5488	0.5117	0.0989	
Quadratic	< 0.0001	0.9539	0.8301	Suggested
Cubic	0.0386	0.9826	0.3525	Aliased

Table S3. Comparison of DEP adsorption performance of cigarette-filter-derived and commercial activated carbons under identical experimental conditions.

Samples	Removal efficiency (%)	Adsorption capacity (mg g^{-1})
AC-300	98.6	27.00
MAC-11	87.0	23.85
CAC-1	99.6	27.29
CAC-2	99.3	27.21

Note: Adsorption capacities were determined under identical experimental conditions and do not represent maximum saturation capacities. For materials exhibiting near-complete DEP removal, the measured capacities were limited by the amount of DEP available in the solution.

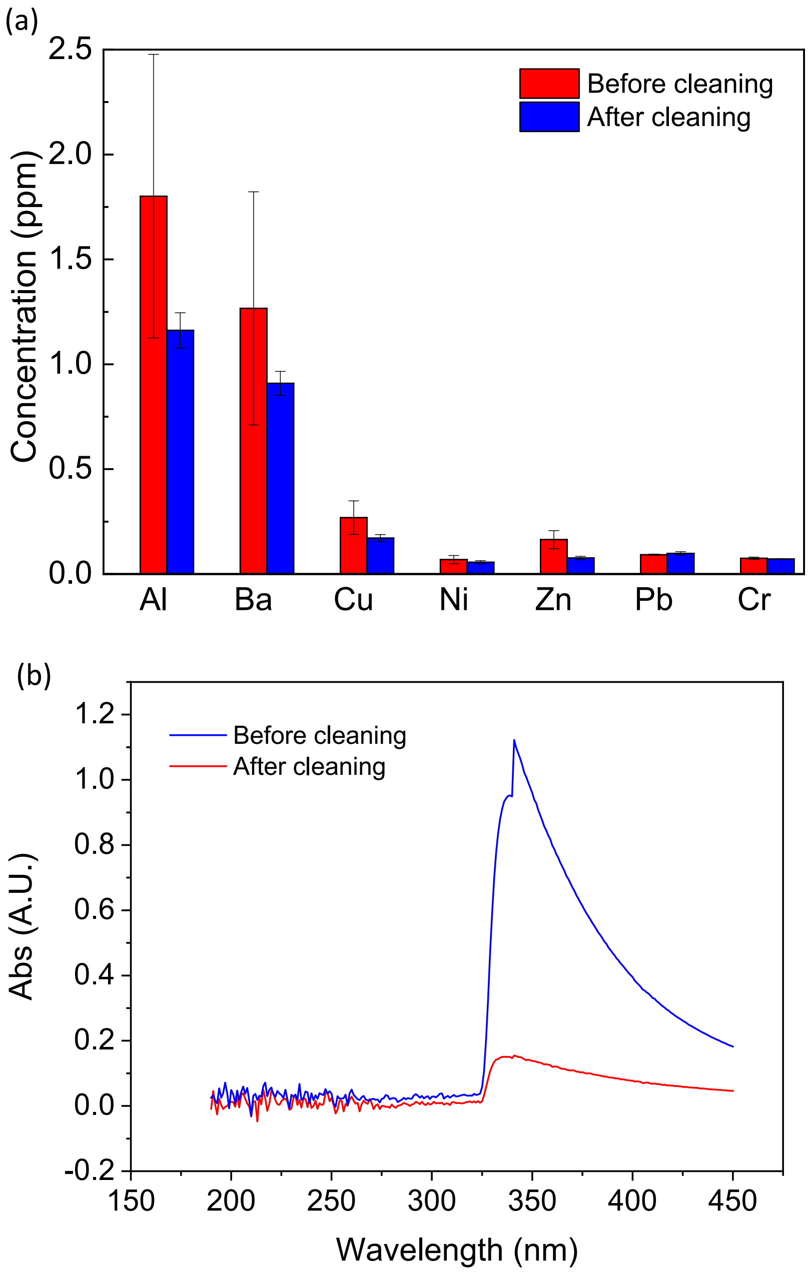

Figure S1. Evaluation of the effectiveness of ethanol washing for discarded cigarette filters: (a) ICP-OES results for selected elements before and after cleaning; (b) UV–Vis absorption spectra obtained before and after cleaning.

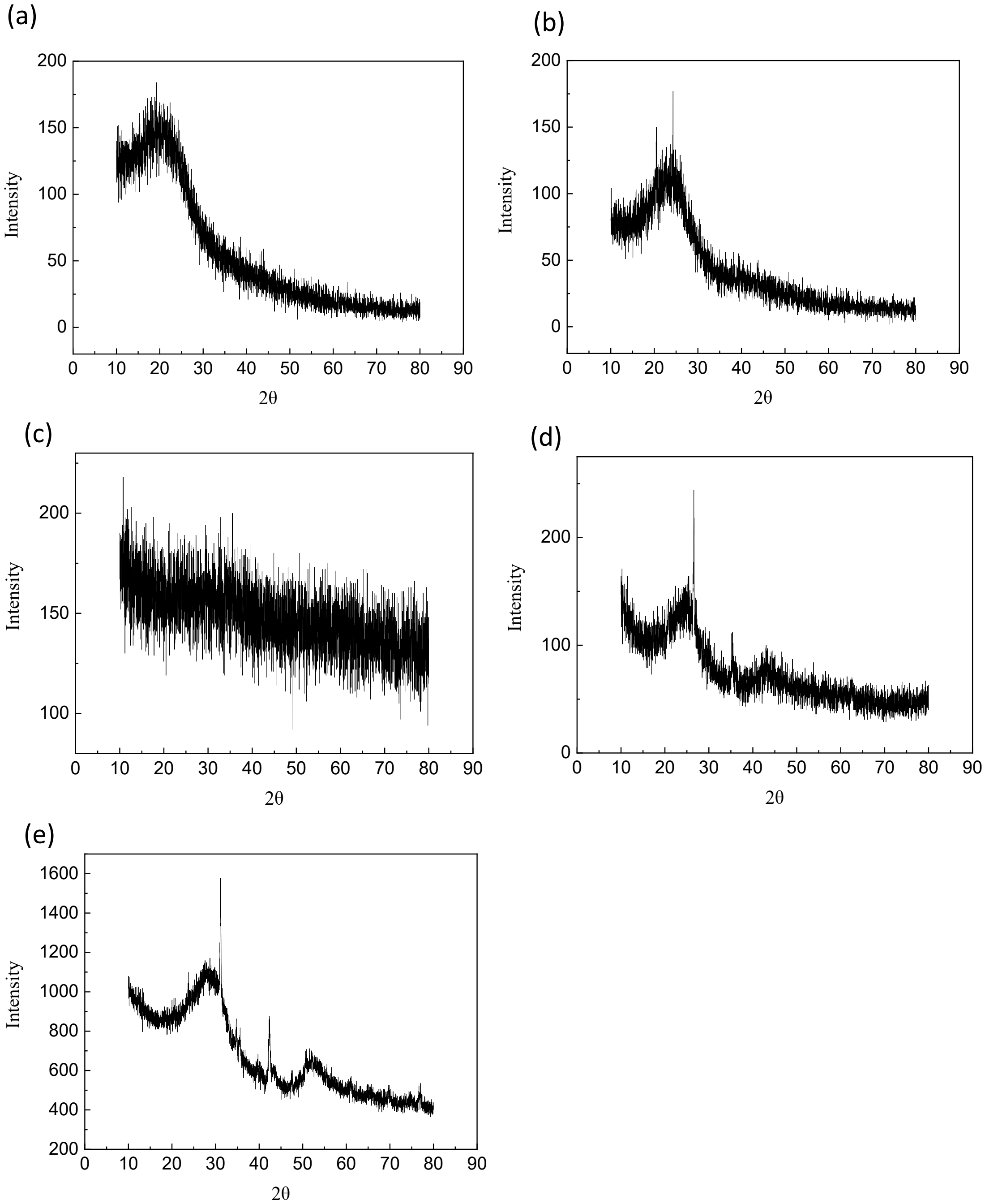

Figure S2. XRD patterns of (a) HTC-CB, (b) AC-300, (c) MAC-11, (d) CAC-1, and (e) CAC-2.

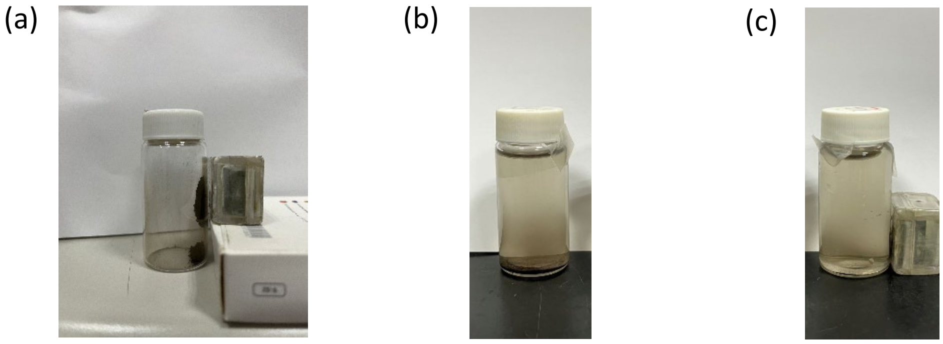

Figure S3. Magnetic response and separation of MAC-11: (a) MAC-11 before dispersion, (b) MAC-11 dispersed in water, and (c) collection of MAC-11 using an external magnet.

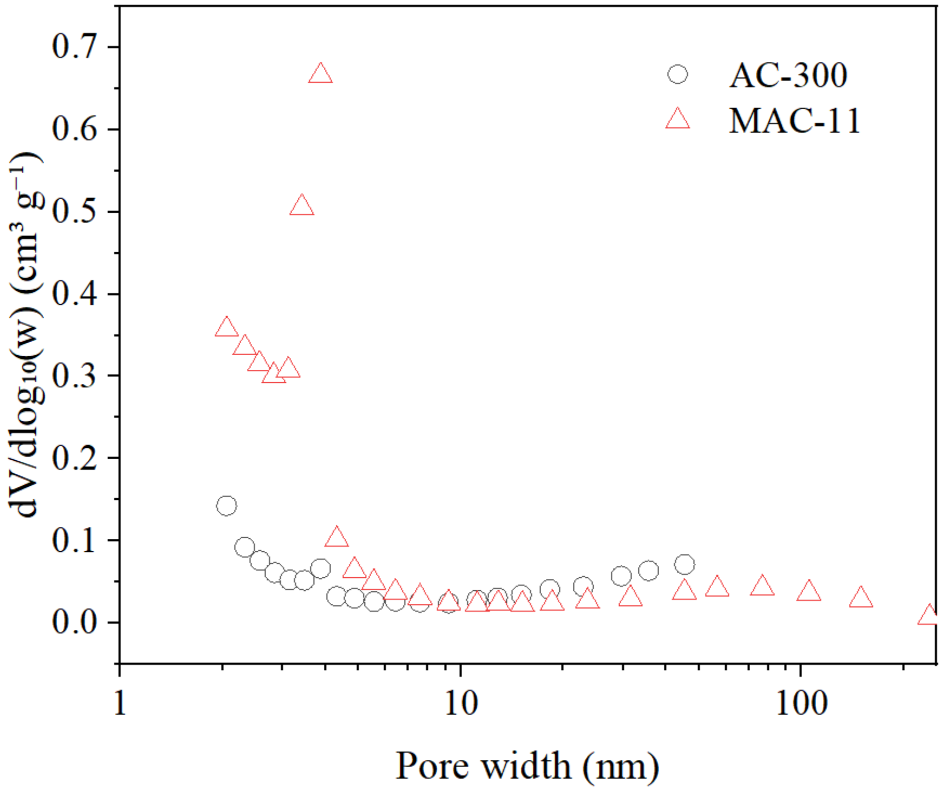

Figure S4. BJH desorption pore-size distributions of AC-300 and MAC-11 obtained from N_2 adsorption–desorption measurements at 77 K. Differential pore volumes are expressed per logarithmic pore-width interval.

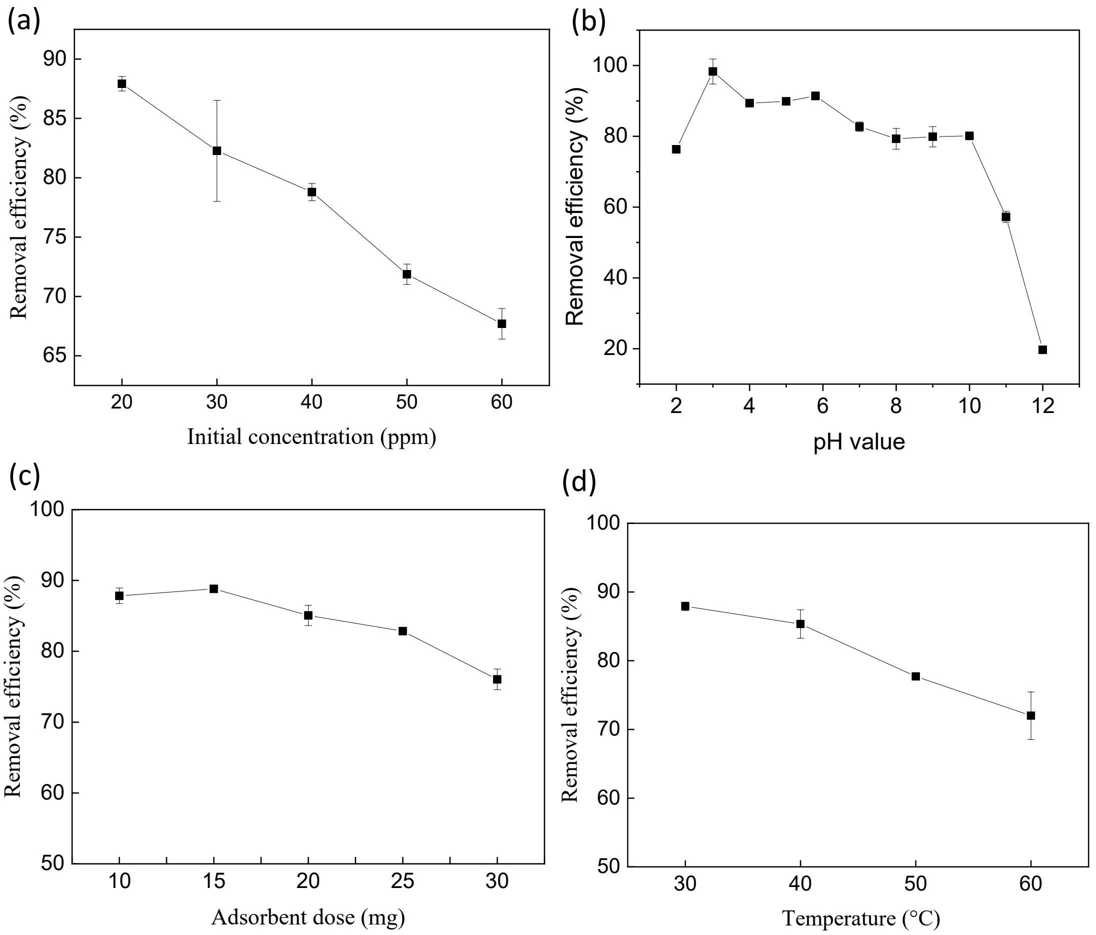

Figure S5. Effects of operating conditions on DEP removal by MAC-11: (a) initial DEP concentration, (b) solution pH, (c) adsorbent dosage, and (d) temperature.

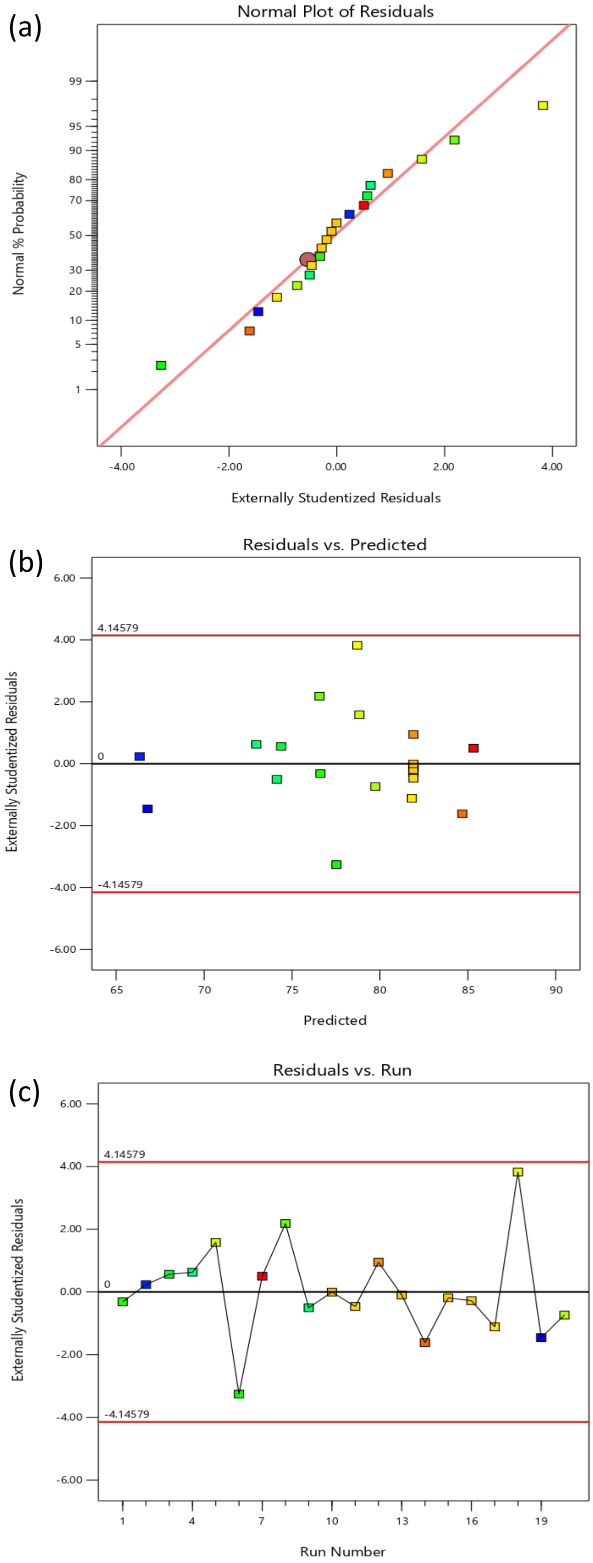

Figure S6. Residual diagnostic plots for the quadratic response surface model: (a) normal probability plot of externally studentized residuals, (b) externally studentized residuals versus predicted DEP removal efficiency, and (c) externally studentized residuals versus experimental run number.

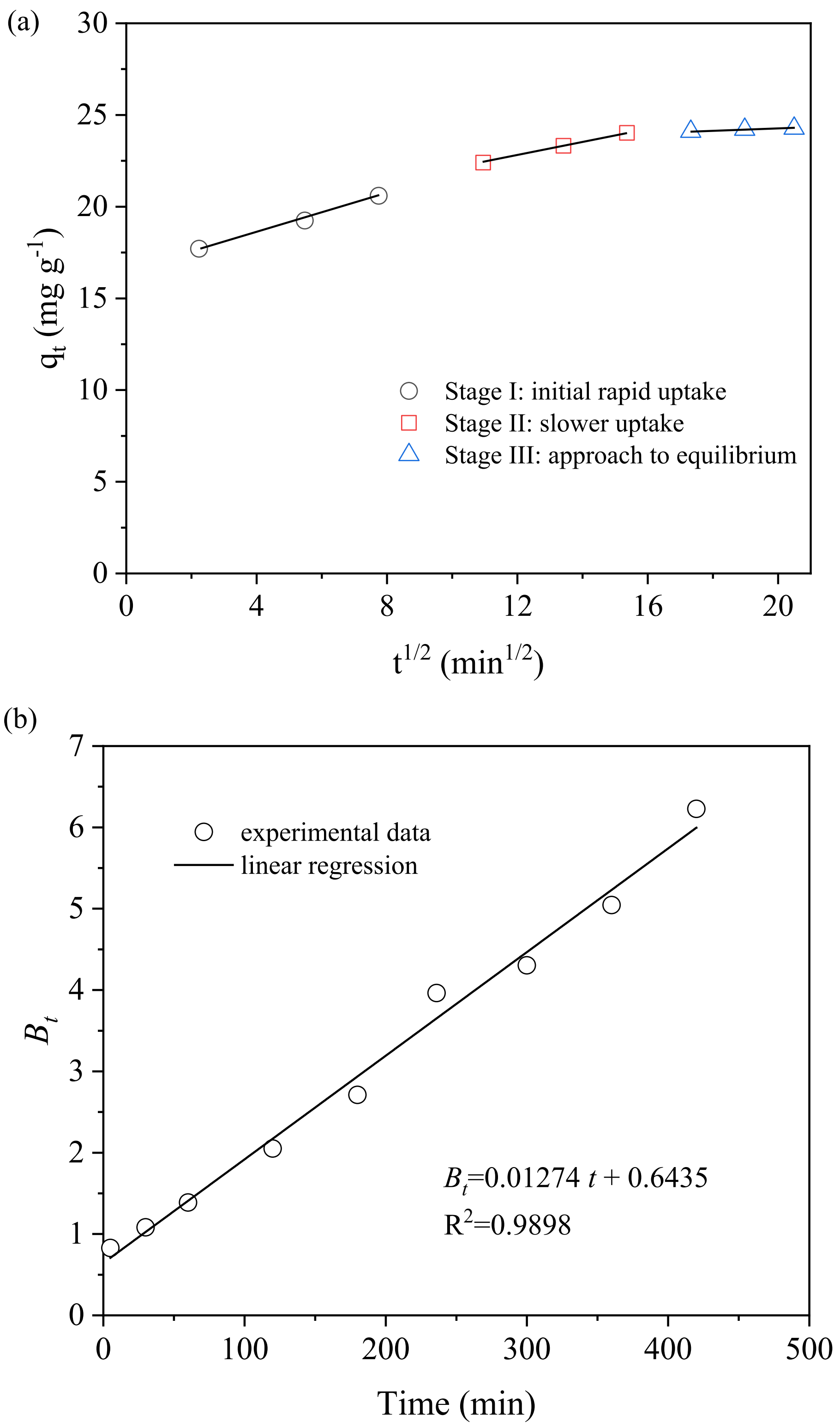

Figure S7. Diffusion-model analysis of DEP adsorption onto MAC-11: (a) Weber–Morris intraparticle diffusion plot showing three apparent uptake regions and (b) Boyd diffusion plot with linear regression.
